# Deep Learning for Pneumonia Detection in Chest X-ray Images: A Comprehensive Survey

**DOI:** 10.3390/jimaging10080176

**Published:** 2024-07-23

**Authors:** Raheel Siddiqi, Sameena Javaid

**Affiliations:** Computer Science Department, Karachi Campus, Bahria University, Karachi 73500, Pakistan; sameenajaved.bukc@bahria.edu.pk

**Keywords:** pneumonia detection, chest X-ray, deep learning, convolutional neural network, COVID-19

## Abstract

This paper addresses the significant problem of identifying the relevant background and contextual literature related to deep learning (DL) as an evolving technology in order to provide a comprehensive analysis of the application of DL to the specific problem of pneumonia detection via chest X-ray (CXR) imaging, which is the most common and cost-effective imaging technique available worldwide for pneumonia diagnosis. This paper in particular addresses the key period associated with COVID-19, 2020–2023, to explain, analyze, and systematically evaluate the limitations of approaches and determine their relative levels of effectiveness. The context in which DL is applied as both an aid to and an automated substitute for existing expert radiography professionals, who often have limited availability, is elaborated in detail. The rationale for the undertaken research is provided, along with a justification of the resources adopted and their relevance. This explanatory text and the subsequent analyses are intended to provide sufficient detail of the problem being addressed, existing solutions, and the limitations of these, ranging in detail from the specific to the more general. Indeed, our analysis and evaluation agree with the generally held view that the use of transformers, specifically, vision transformers (ViTs), is the most promising technique for obtaining further effective results in the area of pneumonia detection using CXR images. However, ViTs require extensive further research to address several limitations, specifically the following: biased CXR datasets, data and code availability, the ease with which a model can be explained, systematic methods of accurate model comparison, the notion of class imbalance in CXR datasets, and the possibility of adversarial attacks, the latter of which remains an area of fundamental research.

## 1. Introduction

Pneumonia is a lung infection caused by various pathogens, including viruses, bacteria, and fungi [1,2]. It affects either one or both lungs and results in the inflammation of lung parenchyma, i.e., the portion of the lung tissue that is responsible for gas exchange, including pulmonary alveoli. The inflammation causes the lung’s alveoli to fill up with pus or fluid, therefore resulting in symptoms like shortness of breath, cough, chest pain, fever, etc. Common pathogens responsible for viral pneumonia include influenza viruses, respiratory syncytial virus, and the SARS-CoV-2 virus, while the most common pathogen of bacterial pneumonia is Streptococcus pneumoniae [3].

Pneumonia is a significant global health threat, leading to high mortality rates worldwide. It is a major cause of death of children (under five years) in developing countries as well as of elderly people (above 65 years) in developed countries [4,5,6]. Recently, the outbreak of SARS-CoV-2 virus has also caused much havoc across the world. Globally, up till the time of writing, there have been more than 702 million confirmed cases of the virus, resulting in around 6.9 million deaths [7]. In many cases, the SARS-CoV-2 virus leads to pneumonia in the infected person [8] and therefore requires the hospitalization of the patient. Since SARS-CoV-2 has a relatively high transmission rate with an R_0_ of around 2.5 for the original variant [9], the medical and healthcare systems of even developed countries have sometimes been overwhelmed by the high number of COVID-19 patients [10]. Due to the aforementioned problems, timely and accurate pneumonia diagnoses are needed for prompt curative treatment, which in turn helps mitigate the pneumonia-associated crisis.

Some common tools to diagnose pneumonia include chest X-rays (CXRs), chest computed tomography (CT) scans, magnetic resonance imaging (MRI) of the chest, chest ultrasound scans, etc. [11]. Despite CXRs’ lower sensitivity for detecting pneumonia compared to that of some other diagnostic tools, like chest CT and chest ultrasound scans [12,13], CXRs are still considered the gold standard for diagnosing pneumonia according to most clinical guidelines globally [14]. Moreover, CXRs are very economical [15], easily accessible, and have very low radiation doses compared to CT scans [16]. For example, a CXR scan delivers only 0.1 millisievert (mSv) of ionized radiation to the patient compared to 7 mSv delivered by a chest CT scan; i.e., one’s exposure to radiation is seventy times higher in the case of the CT scan. Compared to MRIs, CXRs also have some benefits. They have lower costs, are much quicker to perform, and are much more commonly and easily available even in resource-constrained parts of the world. For these reasons, CXRs are the most commonly performed radiological scans in the world [17].

In the context of the COVID-19 pandemic, CXRs enable the rapid triaging of patients during a COVID-19 wave [18]. This is because the gold-standard diagnostic test for SARS-CoV-2 is reverse transcription-polymerase chain reaction (RT-PCR) [19] but this is time-consuming, involves a laborious manual process [20], and also suffers from a low level of sensitivity [20]. The use of CXR-based diagnosis in parallel with RT-PCR (which takes longer) can help prioritize patients and improve survival rates. In addition, portable CXRs ensure patient isolation and help in preventing the spread of the virus [18,21]. All this is possible only because COVID-19 pneumonia has some unique manifestations on CXRs [21], which are different from other forms of pneumonia.

Unfortunately, despite all the aforementioned benefits of using CXRs as a tool to diagnose pneumonia, there is a dearth of expert radiologists [6,15,22], especially in developing countries, that can accurately interpret CXRs; i.e., there is often a serious imbalance between the number of patients and the number of available radiologists. Moreover, since the resolution of CXRs is lower than that of CT and MRI scans, there is always a chance that even an expert radiologist or clinician may miss out some important pattern or manifestation present in a CXR [6]. Computer-aided diagnostic (CAD) tools can help medical staff like radiologists and clinicians in pneumonia diagnosis. Deep-learning (DL)-based methods have been extensively evaluated as an underlying CAD technology for pneumonia diagnosis [1,2,6,8,11,15,18,23,24,25,26].

Over the past decade, DL methods have dramatically improved the state of the art in visual recognition tasks [27,28]. This includes diagnostic accuracy in medical imaging [29], such as pneumonia diagnosis in CXR. Unlike the traditional machine learning (ML) approach, DL methods do not require domain expertise or hand-crafted features. This is because in DL, features are learned from the data directly using a general-purpose learning procedure called backpropagation [27]. This enables automatic end-to-end feature extraction and image classification. A convolutional neural network (CNN) is a type of deep neural network that specializes in visual recognition tasks such as pneumonia diagnosis in CXRs [30]. An important challenge in exploiting CNNs for diagnostic tasks is the scarcity of high-quality correctly labeled large-size medical image datasets. Training on small datasets often leads to problems such as overfitting, poor generalization, etc. Moreover, training on poorly labeled datasets will also likely lead to misleading diagnoses. The public availability of some high-quality CXR datasets [1,31,32,33,34,35] has certainly helped promote research in the area of automated chest disease diagnoses. In some research studies [36,37], generative adversarial network (GAN)-based synthetic CXR generation techniques have been successfully exploited to overcome the problems of overfitting and poor generalization.

In this paper, we review the research carried out in the area of pneumonia identification in CXR images using DL in the last eleven years, i.e., from 2012 to 2023. The year 2012 was chosen as the starting year because deep learning was adopted by the computer vision community as its mainstay after its remarkable performance in the ImageNet Large Scale Visual Recognition Challenge (ILSVRC) in 2012 [27,38]. Unfortunately, from 2012 till 2016, almost all research in this area was carried out using ML approaches [39,40] rather than DL. So, in essence, this paper covers research studies published after 2016. In order to be more relevant to the state of the art, this study mainly focuses on research works published from 2020 onwards. The last search was performed on 30 November 2023, and therefore, this study only considers research published up to this date. The articles were searched in four electronic research databases, namely IEEE Xplore, SpringerLink, ScienceDirect, and ACM Digital Library. This paper provides a comprehensive review and critical analyses of the searched literature.

## 2. Research Questions

Research questions provide motivation and guidance throughout the research process [41]. A research study is normally based on research questions [42]. In this respect, the key research question for this survey paper is as follows: “How can we draw a comprehensive picture of the state of research in the field of pneumonia detection using CXRs using deep learning?” To comprehensively answer this main research question, the following research questions were formulated:

RQ-1: What are the basic concepts behind pneumonia detection using CXRs using DL?

Rationale: Answering this question will establish the theoretical foundation of this research field.

RQ-2: What are the publicly available pneumonia-specific CXR datasets?

Rationale: The answer to this question will involve listing and describing the various publicly available, labelled, and pneumonia-specific CXR datasets.

RQ-3: What are some key statistics in this research area?

Rationale: Answering this question will unveil key statistics in this research area. It will indicate how interest in this field has evolved over the course of time. Moreover, it will also reveal other important statistics, such as the relative frequencies of the commonly exploited methods, the number of studies focusing on COVID-19 pneumonia detection, etc.

RQ-4: What are some of the recent techniques that have been thoroughly evaluated and have yielded promising results?

Rationale: The answer to this question will elaborate on the various recent DL algorithms and architectures involved in solving the problem of pneumonia detection using CXRs.

RQ-5: What are some key issues and challenges in this field?

Rationale: The answer to this question will involve highlighting key challenges and issues that the researchers in this field need to resolve.

## 3. Research Method

An initial search was performed on Google Scholar with phrases like “pneumonia detection in CXR using deep learning” and “COVID-19 detection in CXR using deep learning”. After finding some relevant papers, a list of keywords was prepared, and search venues were selected. The keywords selected were “pneumonia”, “chest X-ray”, “CXR”, “chest radiograph”, “deep learning”, “convolutional neural network”, and “cnn”. The list of keywords was finalized based on the most frequently occurring keywords in the relevant papers. Four electronic research databases [43] were finalized based on their credibility and relevance. They were “IEEE Xplore”, “ScienceDirect”, “SpringerLink”, and “ACM Digital Library”. The following search query [17] was designed based on the selected keywords: “pneumonia” AND (“CXR” OR “chest X-ray” OR “chest radiograph”) AND (“deep learning” OR “CNN” OR “convolutional neural network”).

Along with specifying the above search query, a number of other search filters were also applied to narrow down the search criteria. For example, the subject area or discipline was restricted to “computer science”, and article type was restricted to “research articles”. When this search query along with relevant filters was run on the four selected research databases, a large number of items were returned. The precise number of items returned by each database is given in Table 1 below.

In total, 789 research items were returned by the four research databases. These research items were then short-listed by following strict inclusion/exclusion criteria, which are outlined below:

### 3.1. Inclusion Criteria

All included studies satisfy the following three criteria:

1. Empirical studies that focus on solving the problem of pneumonia detection using CXRs using DL;
2. Studies published in a peer-reviewed journal or conference proceeding;
3. Studies published between 2020 and 2023 inclusively.

### 3.2. Exclusion Criteria

1. If a paper or a publication fell into any of the following categories, it was excluded:
2. All reviews and survey papers;
3. All non-peer-reviewed publications;
4. Short papers less than 5 pages long;
5. Book chapters, as these are usually reviews of a research area;
6. All papers that were scientifically unsound. Scientifically unsound papers include all papers in which methodology is not meticulously presented or in which the hypothesis or the solution is not methodically evaluated;
7. All papers not written in English;
8. Duplicate papers were removed. For example, only a single item was retained if the same item was returned by two or more different databases;
9. Papers focusing on detection of some other chest disease like tuberculosis, pneumothorax, cardiomegaly, etc., in which pneumonia detection is not considered;
10. All papers exclusively exploiting traditional ML approaches;
11. All papers exclusively based on non-CXR modalities like CT scans, ultrasound scans, etc.

Point number 5 of the exclusion criteria states that scientifically unsound papers were discarded. It is vital to be more precise about what constitutes a scientifically unsound paper. For this purpose, quality assessment criteria were formulated. For a paper to be scientifically sound, the answer to all the following questions should be ‘yes’:

1. Are the research objectives clear and well defined?
2. Is the methodology comprehensively explained?
3. Is the proposed solution thoroughly evaluated?
4. Are the limitations of the research clearly stated?
5. Is the research work published in a reputable journal or conference proceeding? For example, any relevant paper published in a journal that has an impact factor less than 3 is discarded.

**Figure 1:**
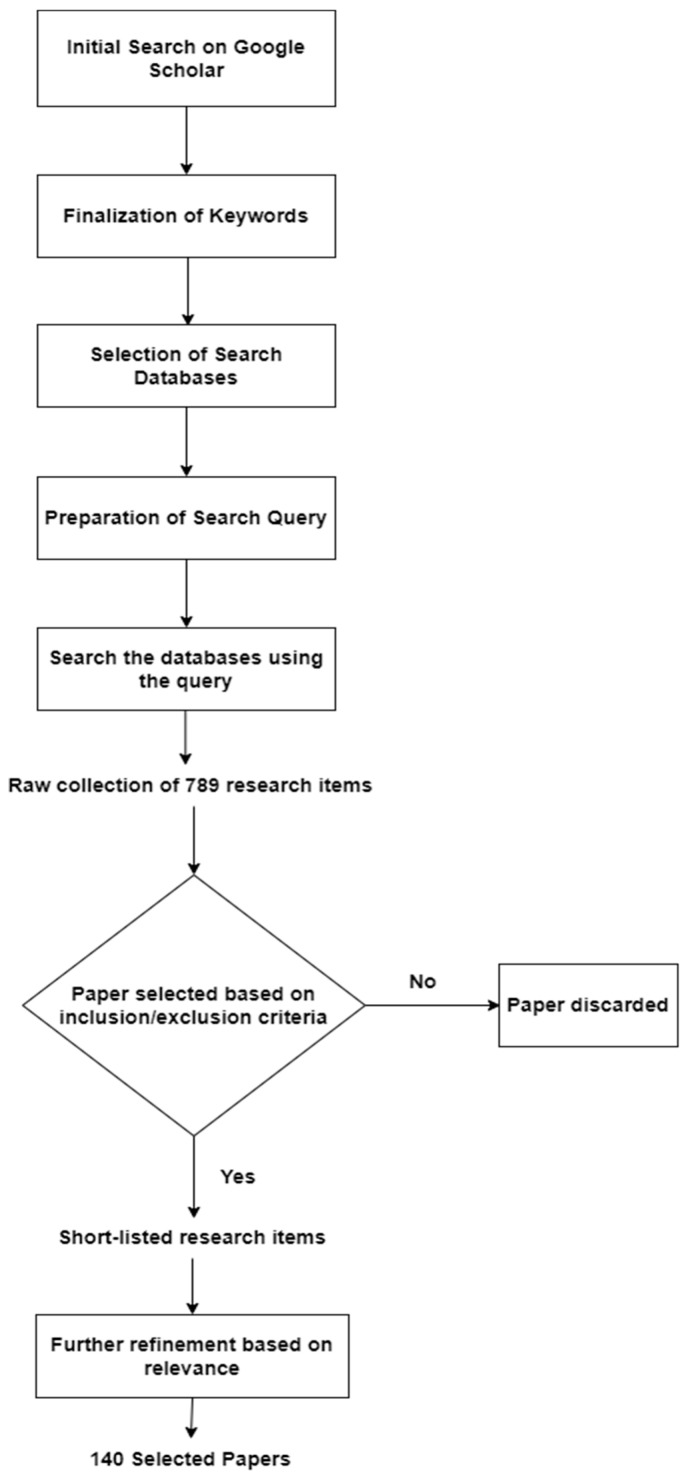
The research paper selection process.

Figure 1 depicts the research paper selection process followed in this study. The first few steps, which resulted in a raw collection of 789 research items, were explained earlier in this section. This raw collection of research items was short-listed and refined by following the above inclusion/exclusion criteria, including the assessment of paper quality. Finally, a limited number of the most relevant papers from the short-listed papers were selected for in-depth review. In total, 140 research papers were selected and reviewed thoroughly to provide an updated and comprehensive picture of this research area.

A Microsoft Excel spreadsheet was created to record and maintain important information about the selected publications. This spreadsheet was updated as more and more selected publications were reviewed. The spreadsheet recorded the following information: *paper title*, *authors*, *publication year*, *journal/conference name*, *keywords*, *publication type* (possible entries: “Journal Paper” or “Conference Paper”), *search database*, *task* (possible entries: “classification”, “localization”, or “both”), *pneumonia type* (possible entries: “covid”, “non-covid”, or “both”), *number of classes* (possible entries: “binary” or “multi-class”), *dataset(s)*, *method*, *evaluation metrics*, *(best) results*, and *critical remarks*.

## 4. Basic Concepts

**Figure 2:**
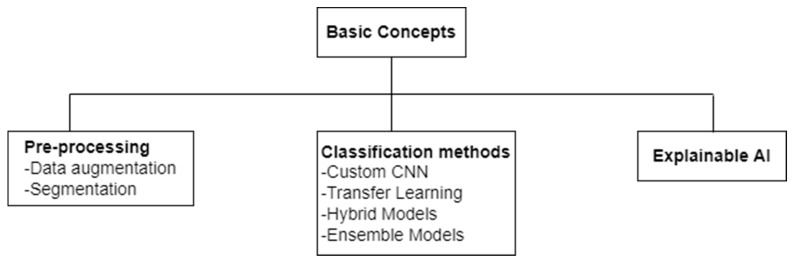
Taxonomy of basic concepts in the area of CXR-based pneumonia detection using deep learning.

In this section, some basic concepts in this area of research are elaborated. These basic concepts are divided into three broad categories: pre-processing, classification methods, and explainable AI. Two pre-processing techniques, namely data augmentation and segmentation, are briefly overviewed in Section 4.1 and Section 4.2, respectively. The classification methods employed for deep-learning-based pneumonia detection using CXRs are divided into four broad categories: CNNs, transfer learning, hybrid models, and ensemble models. The underlying concepts behind these four terms are described briefly in Section 4.3, Section 4.4, Section 4.5 and Section 4.6. Finally, the concept of explainable AI is briefly discussed in Section 4.7. Each section from Section 4.1, Section 4.2, Section 4.3, Section 4.4, Section 4.5, Section 4.6 and Section 4.7 also indicates the pneumonia detection studies that have exploited the concept overviewed. Figure 2 presents the taxonomy of the basic concepts discussed.

### 4.1. Data Augmentation

**Figure 3:**
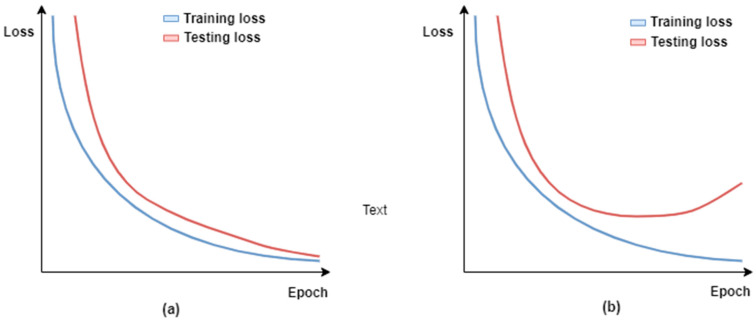
Visualization of training and testing loss over training epochs for two different scenarios: (**a**) the trained model is useful because the validation loss decreases with the training loss; and (**b**) the trained model loses its generalization ability, i.e., overfitted.

Medical image classification tasks, such as pneumonia detection using CXRs, usually suffer from the problem of limited data [44]. This may lead to model overfitting, which means that the trained model lacks generalization ability, i.e., the model performs perfectly on training data but demonstrates a poor performance on test data. In order to build useful models, the test set accuracy should be high and close to the training set accuracy. Figure 3 illustrates the training and validation curves for the two different scenarios: (1) the trained model is useful because the validation loss decreases with the training loss; and (2) the trained model loses its generalization ability, i.e., it fits too closely to the training data, as its validation loss starts to increase over the course of model training. Figure 3a,b illustrate the first and second scenarios, respectively.

There are a few techniques to reduce overfitting, and data augmentation is one of them. Data augmentation has been extensively exploited for pneumonia detection using CXRs [11,45,46,47]. It involves artificially creating new training samples that are realistic variants of the original training samples. This inflates the size of the training set and helps mitigate the issue of limited data. The new training samples are generated either through data warping [48] or synthetic over-sampling [49]. Data-warping augmentations usually involve generating new samples by applying some geometric transformations on existing images. Possible geometric transformations include shearing, scaling, rotating, reflecting, translating, etc. The newly generated images are added to the training set. Even though there are other types of data-warping augmentations, such as color transformations, random erasing [50], adversarial training [51], etc., geometric transformations remain the most widely exploited data-warping approach for medical image classification problems [52]. Data warping preserves the label of the original sample such that the new sample has the same label as the original sample. Data-warping transformations are often used in combination. For example, a new sample may be created by applying a combination of geometric and color transformations on the original image.

In addition to the above data augmentation mechanisms, generative adversarial networks (GANs) [53,54] have also been exploited to augment CXR image data in various studies [37,55,56,57,58]. Jabbar et al. [59] state that a GAN is the most common unsupervised learning model in machine learning. GANs are capable of generating high-quality, synthetic image data at a fast rate and generally perform better than other generative models [59]. If a GAN has been properly trained, the generated synthetic images are indistinguishable from real data samples.

### 4.2. Segmentation

Like data augmentation, segmentation is a pre-processing step applied to CXR images [60]. Segmentation is typically performed to locate objects (e.g., lungs) and boundaries (e.g., lung boundaries) in images (e.g., CXR images) [61]. It enables the extraction of desired regions of interest from CXR images. Manual segmentation is tedious and time-consuming and relies on the expertise of radiologists. DL-based segmentation models have been used to obtain the lung region in CXR images. Narayanan et al. [62] have made use of CXR image segmentation as a pre-processing step. They did this using a DL-based segmentation model called U-Net [63]. Through segmentation, they were able to find out that the shape of the lung is key to differentiating between viral and bacterial pneumonia. The performance of the classification model improved as a result of incorporating segmentation in the process.

A number of DL-based segmentation models exist such as fully convolutional networks (FCNs) [64], U-Net [63], and V-Net [65]. U-Net is the most popular architecture [60]. The following are some of the studies that have exploited segmentation in CXR-based pneumonia detection: [66,67,68,69,70,71].

### 4.3. Convolutional Neural Networks

CNNs are inspired by the brain’s visual cortex, and it is the type of DL model best-suited for computer vision tasks [72] like classification and localization. Even though the concept of CNNs has been around since the 1980s, they were adopted as the mainstay for computer vision tasks after achieving remarkable, state-of-the-art results in the ILSVRC from 2012 onwards [27,38]. This relatively recent, meteoric rise of CNNs is largely due to three key factors: (1) the availability of more sophisticated CNN techniques, (2) the availability of high-performance graphical processing units (GPUs), and (3) the availability of large volumes of training data [60]. Apart from producing higher accuracy levels, another advantage of using CNNs is that they require much less data pre-processing compared with traditional machine learning methods [73]. The following are some of the studies that have designed and evaluated their own custom CNNs for the task of CXR-based pneumonia detection: [71,74,75,76,77,78,79,80,81,82,83,84,85,86].

**Figure 4:**
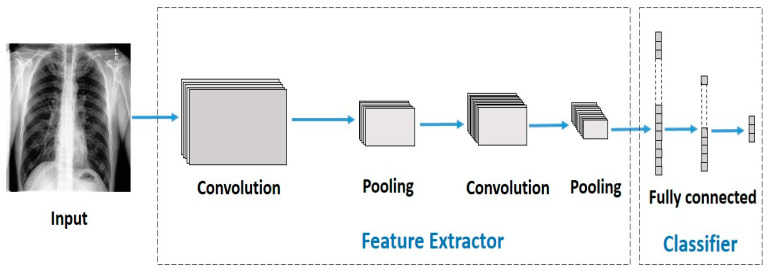
A sample CNN architecture.

CNNs are simply stacked multi-layered neural networks [87]. There are three main layer types in a CNN: (1) the convolutional layer, (2) pooling layer, and (3) fully connected layer. Figure 4 depicts a sample CNN architecture.

### 4.4. Transfer Learning

**Figure 5:**
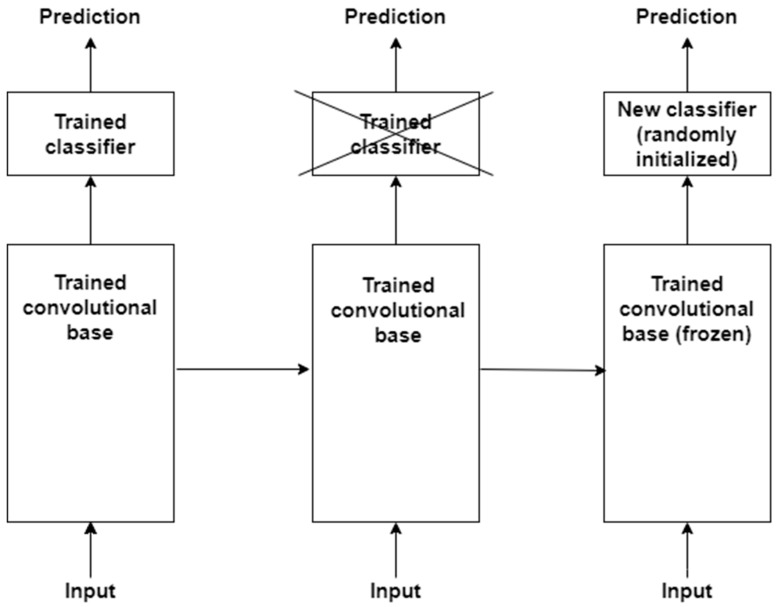
First approach to performing transfer learning is based on training a new classifier on top of a frozen feature extractor, i.e., convolutional base.

**Figure 6:**
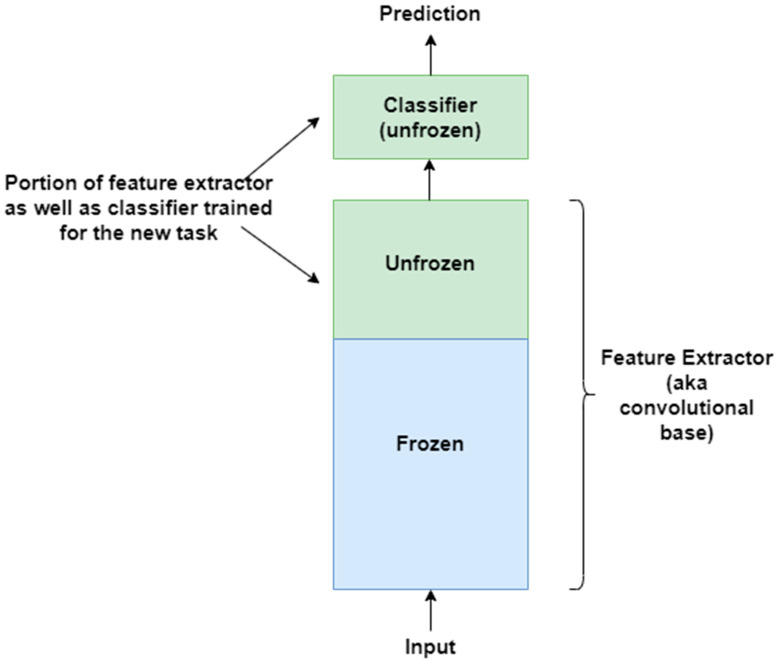
Second approach to transfer learning involves fine-tuning the unfrozen portion of the feature extractor as well as classifier for the new task.

The basic idea behind transfer learning is that a model trained on a large dataset containing images of a particular domain (e.g., natural object images) is repurposed for classifying images of some other dataset belonging to a different domain (e.g., CXR images) [88,89]. The latter dataset is usually a small dataset with a limited number of images [90]. Transfer learning is a very effective technique when there is scarcity of well-labelled data in the target domain but a huge collection of well-labelled data in some other domain. The latter may be in a different feature space or follow a different data distribution. The generic features learned by the model during training on a large dataset are reused for the target task. Therefore, such models do not need to be trained from scratch, saving time and resources [91]. There are two approaches to transferring knowledge from one domain to another [88]. In the first approach, a pre-trained model is treated as a feature extractor, and its weights are kept frozen [92]. The original trained classifier is removed from the top of the feature extractor and is replaced by an untrained, randomly initialized classifier. The new classifier is then trained on top of the frozen architecture for the new task. This approach is depicted in Figure 5. The second approach involves unfreezing part or all of the feature extractor [45,93]. This approach is referred to as *fine-tuning*. The weights of the pre-trained feature extractor are used as a baseline during fine-tuning; i.e., the weights are not randomly initialized. This enables faster convergence during training. The fine-tuning technique is based on the idea that low-level visual features are transferable from one domain to another. In a CNN, low-level visual features are extracted by the initial layers of a feature extractor. Therefore, the initial layers are kept frozen. The last layers of the feature extractor are unfrozen and trained for the new task along with the added classifier. This second approach to transfer learning is depicted in Figure 6. The following are some of the studies that have exploited transfer learning for CXR-based pneumonia detection: [45,47,79,88,91,92,94,95,96,97,98,99,100,101,102].

### 4.5. Hybrid Deep Learning Models

Hybrid models are designed in a way such that they fuse together different neural network architectures and machine learning algorithms to form a single architecture [103]. Such models are meant to solve challenging problems by combining the strengths of various approaches. Fundamentally, hybrid models try to overcome the limitations of a standalone, pure deep neural network by combining it with other neural network architectures and/or machine learning techniques. The following are some of the studies that have exploited hybrid models to solve CXR-based pneumonia detection problems: [11,104,105,106,107,108]. Issues such as performance, efficiency, interpretability, etc., determine the choice of constituent architectures and techniques in a hybrid model.

### 4.6. Ensemble Deep Learning Models

**Figure 7:**
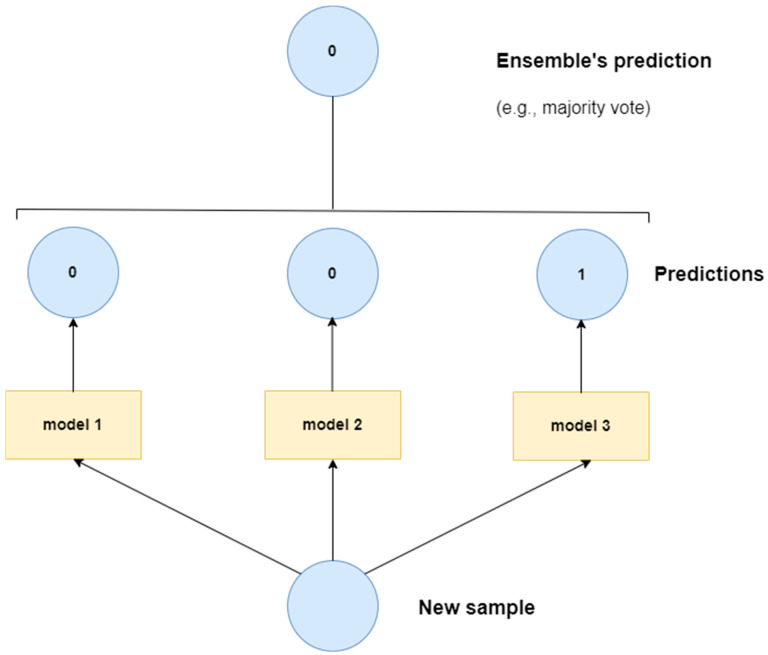
Sample ensemble model.

Ensemble models in deep learning combine various machine learning models, often neural networks, to improve overall performance. By combining the strengths of various models, ensemble methods can generate more accurate and robust predictions. In essence, different individual models work independently to generate their own classification output. The outputs from individual models are combined through some voting system, e.g., a majority vote, to produce the final classification output. The core idea is that averaging or combining the predictions of multiple individual models results in a better overall model than an individual model on its own. Figure 7 depicts a simplified diagram of a sample ensemble model. The following studies have exploited some form of an ensemble model for CXR-based pneumonia detection: [109,110,111,112,113,114,115].

### 4.7. Explainable Artificial Intelligence (XAI)

Deep learning methods have shown great promise for pneumonia detection using CXRs. However, medical experts and physicians still lack confidence in deep learning methods, primarily because of their black-box nature [116]. Since human lives are at stake, there are numerous issues that need to be addressed [117]: Can the reasons for a correct diagnosis be explained? How can these methods be exploited for further system improvement? Who is responsible if things go wrong? XAI is a research field that aims to address these issues by making DL systems more transparent and understandable to humans [118]. XAI has been extensively exploited for DL-based pneumonia detection using CXRs [8,47,71,75,78,80,88,92,119,120,121,122,123,124,125,126]. Despite extensive use of XAI in CXR-based pneumonia detection systems, a recent study [127] has argued that the explanations produced by XAI are either unreliable or only offer superficial explanations for individual cases. The study concludes that XAI explanations are often misleading with respect to individual decisions.

## 5. Datasets

Medical imaging datasets have evolved as the foundation of contemporary diagnostic research. These datasets serve as the essential building blocks upon which machine learning and deep learning algorithms are created and tested in the context of pneumonia and COVID-19 diagnosis. The current study explores a wide range of publicly accessible CXR image datasets. These datasets, around 10 in total, are essential for the creation and verification of algorithms for the detection of COVID-19 and pneumonia. They act as the standard against which the effectiveness of different algorithms, which are highlighted in the reviewed publications, is assessed. This collection includes datasets that are specifically devoted to COVID-19 and only contain CXR images associated with the virus, while other datasets present a more varied selection of images, including those of healthy patients as well as instances of CXRs showing manifestations of viral or bacterial pneumonia. The research community’s overarching goal of developing flexible algorithms capable of detecting pneumonia among a variety of radiographic images is reflected in the use of such a varied group of datasets. As part of our thorough analysis of a few key datasets in this study, we also give quick download links in Table 2, which will make it easier for researchers to access the data and promote more investigation in this important area.

Table 2 provides a review of various CXR datasets commonly used in tasks such as pneumonia detection and COVID-19 diagnosis. It contains a variety of datasets, each distinguished by the number of images, the number of classes, and the specific classes contained within them. This table also includes references to scholarly papers that have used these datasets, making it a significant resource for scholars and practitioners in the field of medical image analysis. These datasets jointly aid in the creation and validation of deep learning models, improving the accuracy and efficiency of chest X-ray analysis and enabling the early detection of pneumonia, particularly COVID-19, which is critical in clinical settings.

Table 2 presents a comprehensive assessment of significant public CXR datasets used for pneumonia and COVID-19 detection. Each dataset’s distinct characteristics make it a significant resource for deep learning researchers. This study will help researchers choose the best dataset for their research aims and computational requirements.

## 6. Key Statistics

In the present review, we explored a total of 262 studies that utilized deep learning architectures to detect pneumonia, especially distinguishing between COVID pneumonia, non-COVID pneumonia, or both. Convolutional neural networks (CNNs), including customized CNNs, transfer learning, ensemble models, and hybrid models, were the focus of our research. We also looked into the use of generative adversarial networks (GANs), explainable AI, and vision transformers, among other things. In addition to the various models, we analyzed multiple datasets related to pneumonia identification via X-ray imaging and consulted various survey papers to acquire a thorough understanding of the present landscape.

Specifically related to non-COVID and COVID pneumonia, 140 papers have been reviewed in this study. As Table 3 presents, out of these 140 papers, 70 focus on both COVID and non-COVID pneumonia, while 48 consider only COVID pneumonia and 22 consider only non-COVID pneumonia. In recent times, there has been more focus on COVID pneumonia and this is obviously due to the COVID-19 pandemic.

**Figure 8:**
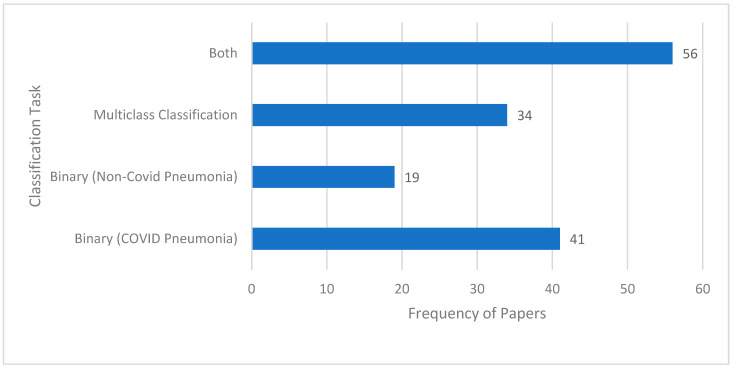
Frequency of papers for the two types of classification tasks.

Another aspect of the reviewed research studies is that they are based on a binary classification task, on multiclass classification, or on both. In the context of this paper, binary classification involves classifying a CXR image into one of the two mutually exclusive classes, e.g., “pneumonia” class or “normal” class. Multiclass classification, in contrast, classifies a CXR image into a class out of more than two possible classes. An example of multiclass classification is a “normal” vs. “viral” vs. “bacterial” vs. “COVID” classification task. Figure 8 illustrates the frequency of papers focusing on each of these classification tasks. It can be seen that there are many studies that consider both classification tasks. Examples of binary and multiclass classification tasks are given in Table 4.

As a detailed overview of the many methodologies used to diagnose pneumonia in chest X-ray (CXR) pictures, the techniques have been grouped methodically into four key categories, demonstrating the expanding landscape of diagnostic techniques.

As indicated by the references [71,74,77,80,81,83,85], the most common technique seen in the provided investigations is the use of custom-designed convolutional neural networks (CNNs). These customized CNNs show promise in catching subtle patterns within CXR pictures for accurate pneumonia detection. Further, the application of transfer learning is closely followed, as evidenced by the following references: [94,95,96,99,100,101]. Transfer learning applies pre-trained models from big datasets to the specific problem of pneumonia identification. This method capitalizes on the knowledge gathered from unrelated tasks, increasing the model’s efficiency.

**Figure 9:**
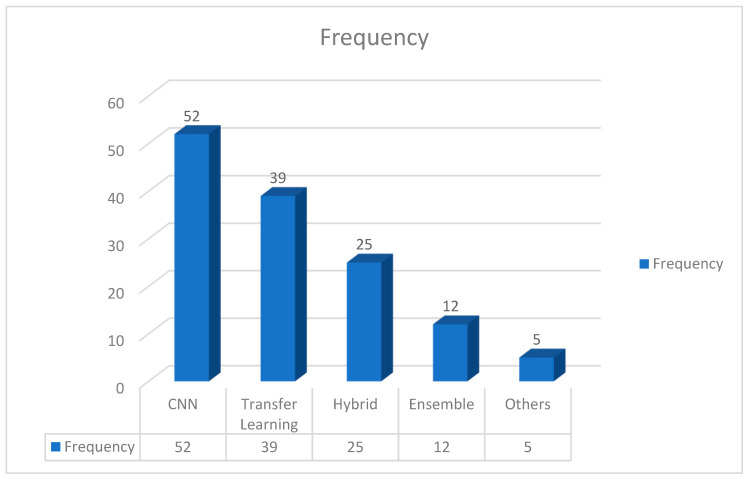
Various methods employed for pneumonia detection using CXR images.

In addition to these main methodologies, other research, such as the references [11,46,108,185,186], investigate the possibilities of hybrid models. Hybrid models combine multiple distinct models to produce higher categorization results. Notably, these constituent models collaborate on decision making by feeding their outputs into one another, resulting in a synergistic impact. Furthermore, as stated by the references [119,128,184], ensemble approaches provide another path for pneumonia diagnosis. Ensemble models, as opposed to hybrid models, are made up of independent constituent models. Each model generates its own classification output, and the final diagnosis is determined by a vote method. This ensemble strategy takes advantage of the diversity of different models, resulting in more robust and dependable predictions. Figure 9 demonstrates the adaptability of pneumonia detection tactics by highlighting the frequency of custom-designed CNNs, the usefulness of transfer learning, and the possibility of hybrid and ensemble models in improving diagnostic accuracy. Figure 9 also depicts the distribution of approaches for detecting pneumonia in chest X-ray (CXR) pictures. With a frequency of 52, custom-designed convolutional neural networks (CNNs) emerge as the most common technique, demonstrating its broad use and efficacy in capturing subtle patterns within CXR images. With a frequency of 39, transfer learning is close behind, suggesting its popularity in modifying pre-trained models for pneumonia diagnosis. Hybrid models, which combine several models for superior categorization, are used 25 times, demonstrating the growing interest in synergistic model combinations in research. Ensemble methods are utilized 12 times, demonstrating their contribution to robust forecasts by utilizing independent constituent models with a voting system. Furthermore, a category called “Others” with a frequency of five includes less frequent or alternative procedures, implying that the field is still exploring new approaches. This quantitative analysis sheds light on the current trends and research priorities in pneumonia detection techniques.

**Figure 10:**
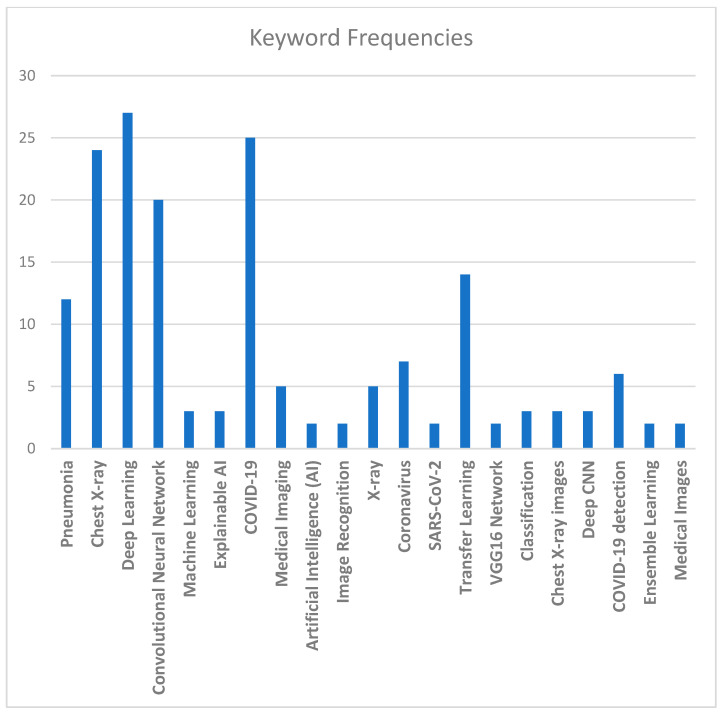
Common keywords and their relative frequencies.

Another aspect used to evaluate studies statistically is commonly occurring keywords in the relevant literature and their relative frequencies, which are given in Figure 10. These keywords can serve as a guide to researchers while searching the related literature. Some of the most commonly used keywords, in order of their significance, are as follows: “Deep Learning”, “COVID-19”, “Chest X-ray”, “Convolutional Neural Network”, “Transfer Learning”, and “Pneumonia”.

**Figure 11:**
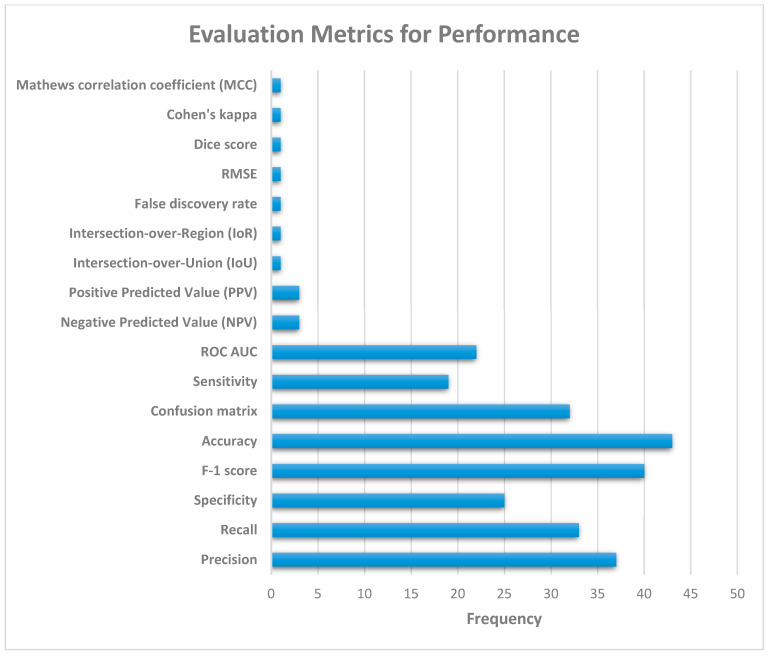
Performance evaluation metrics for pneumonia detection systems.

Figure 11 illustrates the various evaluation metrics used to evaluate the performance of pneumonia detection systems. Based on Figure 11, the most commonly used evaluation metrics (in order of their relative usage) are as follows: accuracy, F-1 score, precision, recall, confusion matrix, specificity, ROC AUC, and sensitivity.

Various deep learning architectures were investigated in this comprehensive analysis of several studies concentrating on pneumonia diagnosis, specifically discriminating between COVID and non-COVID pneumonia. Convolutional neural networks (CNNs) were the focus of the investigation, which included customized CNNs, transfer learning, ensemble models, and hybrid models. We also looked into generative adversarial networks (GANs), explainable AI, and vision transformers. For a comprehensive grasp of the existing landscape, we combined data from different pneumonia diagnosis datasets via X-ray imaging and referenced survey studies. The review of 140 papers on non-COVID and COVID pneumonia found a growing emphasis on COVID pneumonia, which was likely affected by the ongoing pandemic. This study also looked at classification tasks, distinguishing between binary and multiclass classifications and providing examples. A statistical review also emphasized the prevalence of frequent keywords in the literature, assisting researchers in accessing relevant studies. Finally, the paper covered the many assessment measures used in analyzing the performance of pneumonia detection systems, highlighting metrics such as accuracy, F-1 score, precision, recall, confusion matrix, specificity, ROC AUC, and sensitivity. The data shown here visually illustrate the distribution and patterns discovered in the examined studies, providing useful insights into the methodology and evaluation criteria that shape the current landscape of pneumonia detection research.

## 7. Current Trends

The detection of pneumonia using chest radiographs or X-rays, specifically the distinction between COVID and non-COVID patients, is a rising area of research. Deep learning approaches have emerged as critical tools for improving the precision and efficacy of various diagnostic processes.

Convolutional neural networks (CNNs) are the foundation of this landscape, acclaimed for their ability to detect subtle patterns in photos. Their application in the analysis of visual data, such as chest X-rays, has proven useful in the identification of pneumonia. CNN-based architectures extract essential information from radiographic images, allowing for the detection of minor subtleties suggestive of various pneumonia types, including those linked with COVID-19. Furthermore, transfer learning has emerged as a strong paradigm, capitalizing on pre-trained models produced on large datasets such as ImageNet. Researchers use previously learnt features by fine-tuning these models on pneumonia-specific datasets, reducing computational costs and data requirements. This method considerably improves the model’s capacity to generalize and categorize pneumonia cases. Correspondingly, ensemble learning methods reinforce this environment even further by combining outputs from various models or utilizing multiple architectures. This combination improves classification accuracy by combining the collective intelligence of multiple models. Ensemble approaches efficiently leverage the capabilities of various models, resulting in increased precision and robustness in pneumonia classification, including the distinction between COVID and non-COVID cases.

Hybrid techniques, complementing these approaches, combine disparate methodologies or other data sources, such as clinical records or laboratory findings, with chest radiographs. This combination improves the comprehensiveness and dependability of diagnostic systems. These strategies aim to increase diagnosis accuracy and provide a more holistic view of pneumonia by combining numerous information sources, allowing for the accurate categorization of COVID and non-COVID pneumonia patients. Deep learning developments, aided by the emergence of larger and more diversified datasets, hold excellent potential for further improving the precision and dependability of pneumonia detection systems. These developments have the potential to catalyze advances in respiratory illness diagnosis, ultimately improving patient care and treatment techniques.

This field’s research focuses not only on accurately discriminating between COVID and non-COVID pneumonia, but also on tackling issues such as data scarcity, class imbalance, and the interpretability of deep learning models in medical situations. Deep learning developments, as well as the availability of larger and more diversified datasets, are projected to improve the accuracy and reliability of pneumonia detection systems, greatly contributing to the diagnosis and treatment of respiratory infections. Table 5 provides the current trends and some of the latest research methodologies for pneumonia detection and classification.

Table 5 summarizes the outcomes with respect to methodology. Specialized convolutional neural networks provide better accuracies; for example, the authors of [187,188] used specialized network topologies, such as DCNN and Enhanced CNN+ResNet-50, to achieve 96.09% and 92.4% accuracies, respectively. They emphasized the need of preprocessing techniques such as intensity normalization and certain network designs in achieving acceptable accuracies. Further, transfer learning architectures such as VGG-19, CDC_Net, and DenseNet201 were used in studies [143,189,190] to achieve high accuracies ranging from 96.48% to 99.39% for multi-classification tasks. These demonstrated the value of using pre-trained models for feature extraction and classification. On the other hand, ensemble learning approaches have been employed in [133,191,192,193]. These studies combined numerous models or techniques, including convolutional networks, self-attention mechanisms, EfficientNet, and stacked ensembles, and achieved accuracies ranging from 98% to 99.21%. These approaches stressed the use of communal knowledge or the selection of optimal features to increase performance. The studies of [193,194] used hybrid algorithms that combined transfer learning and deep learning (AlexNet, C+EffxNet). They achieved 97.9% and 99.2% accuracies while reducing model complexity and improving decision support systems through feature merging. Subsequently, other specialized architectures like transformers [163,195] have achieved accuracies of 99.13% and 94.96%, respectively. These focused on reducing computing complexity and increasing accuracy.

**Table 5:** Current trends in pneumonia detection.

Ref./ Year	COVID/Non COVID Pneumonia	Binary/Multi Classification	Methodology	Results	Contribution	Research Gap
[189] 2023	Both	Multi Classification	Transfer Learning, VGG-19+CNN	96.48%	Good accuracy	Powerful segmentation models for precise ROI identification are required.
[194] 2023	Non-COVID Pneumonia	Binary Classification	Hybrid technique	97.9%	Simplified the model by reducing advance feature extraction.	Unbalanced data distribution
[143] 2023	Both	Multi Classification	CDC_Net	99.39%	Structured noise reduced	NA
[191] 2023	Non-COVID Pneumonia	Binary Classification	Ensemble CNN+ Transformer Encoder	99.21%	Self-attention mechanism provided more accurate results	Annotated text data required
[195] 2023	COVID Pneumonia	Binary Classification	Multi-level self-attention mechanismTransforme r	99.13%	Reduced computing complexity to increase the efficiency of the recognition process.	Multi-classification model could be enhanced
[187] 2023	Non-COVID Pneumonia	Binary Classification	DCNN	96.09%	Impactful preprocessing techniques	NA
[190] 2023	Both	Multi Classification	DenseNet201	99.1%	DenseNet provides collective knowledge	NA
[192] 2023	Both	Multi Classification	Ensemble Learning (EfficientNet)	98%	NA	Attention-based feature fusion may reduce complexity
[188] 2023	Non-COVID Pneumonia	Binary Classification	Enhanced CNN+ResNet-50	92.4%	NA	NA
[193] 2023	Non-COVID Pneumonia	Multi Classification	Hybrid deep learning model (C+EffxNet)	99.2%	The application of feature merging improved the decision support system.	To better predict chest infections, more classes can be included
[133] 2023	Non-COVID Pneumonia	Multi Classification	Stacked ensemble learning	98.3%	Reduced features are promoted to the stacking classifier.	Preprocessing and reinforment learning can improve results
[163] 2023	Both	Multi Classification	Vision Transformer (PneuNet)	94.96%	Binary pneumonia classification model achieved 99.29% accuracy	Channel-wise transformer encoder can enhance results

Considering Table 5, each study’s research gap emphasizes critical areas for advancement, such as segmentation refinement, addressing data imbalance, noise reduction, interpretability enhancement, computing efficiency, and the exploration of preprocessing and feature extraction techniques for more robust and reliable pneumonia detection models. A study [189] has shown an impressive accuracy in diagnosing six types of COVID and non-COVID pneumonia. It did, however, identify an important research gap: the need for more robust segmentation models. These models would make it easier to identify regions of interest (ROIs) on chest radiographs. By improving segmentation capabilities, the model could more precisely target affected areas, ultimately refining the classification process for higher levels of precision and dependability. A study [194] shed light on the issue of data imbalance in pneumonia datasets. It emphasized the possible application of generative adversarial networks (GANs) to address this issue, with the goal of balancing the representation of multiple classes, particularly in cases with few instances. Furthermore, the study recommended investigating capsule networks due to their ability to perform successfully with fewer training data. This indicates a research void in investigating approaches that require fewer data while maintaining robust performance in pneumonia classification tasks. Despite obtaining an outstanding accuracy and controlling the impact of structured noise, a study [143] highlighted a critical research need. It stressed the importance of further refining segmentation models to improve the identification and delineation of abnormal characteristics in chest radiographs. Furthermore, decreasing the negative impact of structured noise is a critical area for more refined and noise-resilient classification models. A study [191] underlined the importance of improving interpretability while attaining a high accuracy using a self-attention technique. When annotated text datasets become accessible, they suggest including textual explanations alongside visual heatmaps. Furthermore, the study emphasized the importance of improving feature extraction processes in order to improve model interpretability and performance. Further, a study [187] indicated the necessity of a broader investigation of preprocessing techniques. Another study [163] raised issues about interpretability after reaching a great accuracy in binary pneumonia categorization. It underscored the proposed PneuNet model’s black-box character and the need for more representative feature extraction methods. The study advised for improving the channel-wise transformer encoder inside the model architecture to increase interpretability and feature representation.

The findings from numerous research in pneumonia classification highlight the importance of Transformer designs in improving detection model accuracy and efficiency. Several research have used Transformer models or recommended their prospective application across numerous techniques and classification tasks (binary and multi-class), emphasizing their importance in this sector. Application of Transformer models for pneumonia detection in CXR images has the following salient points:

- Transformer-based designs have been successfully employed in both multi-class and binary classification settings in studies. These models equipped with self-attention mechanisms have shown remarkable accuracies ranging from 94.96% to 99.39%.
- Several studies have shown that transformers minimize computational complexity without sacrificing accuracy. They promote efficient recognition processes, which are critical in medical applications in which prompt diagnosis is required.
- Despite its usefulness, some research using transformer models has presented interpretability difficulties. To improve interpretability and feature representation, there is a clear need to improve feature extraction approaches inside transformer designs.
- Transformers have demonstrated adaptability and resilience by being successfully deployed in both COVID and non-COVID pneumonia detection across many classification challenges.

We propose that vision transformers (ViTs) are the most suited method for future work in the area of pneumonia detection using CXR images. ViTs have demonstrated exceptional potential in a variety of image-processing tasks, including natural images. They have shown a competitive performance when compared to that of convolutional neural networks (CNNs), which have long been the go-to architecture for image processing.

ViTs have the following advantages for X-ray image analysis:

1. Handling Global Information: ViTs capture global relationships inside an image. This understanding of context and relationships between different regions may be especially important in medical imaging, for which the context of anomalies in an X-ray may be critical for diagnosis;
2. Fewer Parameters: Because ViTs can process pictures without depending on complex convolutional procedures, they may require fewer parameters than typical CNNs, making them more efficient;
3. Transfer Learning: ViTs have demonstrated promise in transfer learning. Pre-trained ViT models developed on large-scale datasets can be fine-tuned on smaller medical datasets, which is especially useful when labeled medical data are limited.

However, there are challenges as well, which can provide a clear future direction to address theoretical gaps:

1. Data Efficiency: ViTs frequently require significant amounts of data for training, and collecting labeled datasets in medical imaging can be difficult due to privacy concerns and data scarcity;
2. Computational Needs: Training ViTs can be computationally demanding, necessitating significant resources and effort;
3. Interpretability: Understanding why a ViT makes a certain decision may be more difficult than with typical CNNs, which may be a worry in essential applications such as medical diagnosis.

Transformers are emerging as the most potential option for future pneumonia detection due to a number of compelling reasons. For initial reasons, their ability to achieve high accuracies—ranging from 94.96% to 99.39%—shows that they are effective across a variety of classification assignments [163,195]. Transformers excel in capturing complicated global dependencies among images, which is crucial for detecting subtle irregularities in chest X-rays that traditional CNNs may miss. Transformers also minimize computational complexity by exploiting self-attention techniques, allowing them to process images faster than traditional CNN systems [195]. This efficiency is critical in medical applications in which speedy and precise analysis is required.

Furthermore, transformers are highly effective in transfer learning scenarios. Pre-trained models from big datasets, such as ImageNet, can be fine-tuned on smaller, more specialized medical datasets, which is especially useful considering the scarcity of annotated medical pictures. This ability to adapt and generalize from large-scale pre-training to specific medical tasks overcomes the prevalent problem of data scarcity in medical imaging [163].

To address the challenges of interpretability and data efficiency, these aspects are being continually improved. The ability of transformers to be integrated with hybrid models or with approaches such as transfer learning and ensemble learning increases their robustness and versatility. These characteristics make transformers an excellent choice for further research and development, promising improvements in both diagnostic accuracy and operating efficiency for pneumonia detection.

## 8. Discussion

This section discusses the key issues and challenges faced in the area of CXR-based pneumonia detection using deep learning.

### 8.1. Biased Datasets

**Figure 12:**
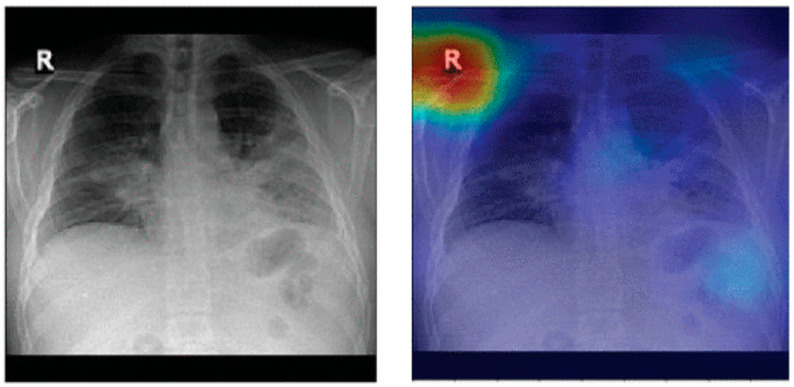

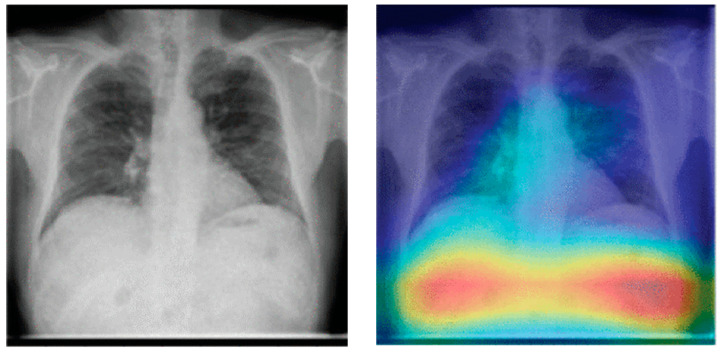
Lung heatmaps generated through Grad-CAM (all images taken from [121]).

It has been shown that pneumonia detection models tend to focus on irrelevant features when classifying CXR images. A detailed bias analysis has been presented in [121]. The classification network used in that study was VGG16 [196]. The network was pretrained on an ImageNet dataset [197], and then, using transfer learning, the network was trained and fine-tuned on domain data. Figure 12 depicts some CXR images that were fed to the model. The figure also shows the corresponding heatmaps generated using Grad-CAM [198]. The heatmaps highlight areas of the image that are critical in the model’s prediction. It can be seen that for both the generated heatmaps in Figure 12, the highly activated regions (denoted in red) are outside the boundaries of the lung tissue. This indicates that features of the lung tissue have not been utilized in the classification decision. The study [121] suggests that this behavior demonstrated by the classification models is due to the inherent biases present in the publicly available pneumonia and COVID-19 CXR image datasets [31,34,158].

These dataset biases could be due to a variety of reasons. An example scenario which can lead to such biases is when a specific CXR machine is used for a group of subjects with a lower chance of disease and a separate CXR machine is used for another group of subjects with a high chance of disease. This may happen when patients highly suspicious of a contagious disease, like COVID-19, are sent to some other area of hospital or even to some other healthcare center for CXR scanning in order to avoid spreading the disease. If one of the two machines generates a white rectangular box at the top right corner in each CXR image and the other one does not, then the model may learn to classify images based on this simple feature rather than true class features.

Another possible scenario is that there is an imbalanced dataset; i.e., there are too few images for one class and a lot more for the other class, e.g., too few COVID-19 CXR images and much more normal CXR images. In order to balance the dataset, COVID-19 samples are included from different data sources. Each of these data sources is likely based on different equipment as well as acquisition protocols. In other words, the samples of the two classes are from different sources. Therefore, the resulting dataset is inherently biased. Any classifier trained on such a dataset may learn to classify CXR images based on source-specific features rather than true class features.

In addition to the acquisition site specific features, the demographic traits of the subjects can be significant confounding factors [199]. For instance, remix datasets that combine adult COVID-19 patients with non-COVID-19 controls from the Guangzhou pediatric dataset (ages 1–5 years) [1] can result in models that erroneously link anatomical features associated with age to the diagnosis.

**Figure 13:**
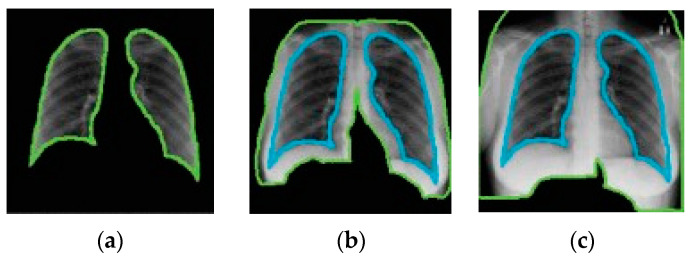
Example of background expansion (all images taken from [121]). From (**a**–**c**) the background is gradually expanded.

Catalá et al. [121] have experimented with background expansion and lung exclusion in CXR images. Background expansion means a background is gradually added to initial lung-only images. Figure 13 depicts an example of background expansion. Ideally, the AUC ROC should not be affected by background expansion. However, empirical results obtained indicate a significant AUC ROC increase when a background is expanded in two of three datasets used. This means that features outside the lung area often play a critical role in correct predictions.

**Figure 14:**
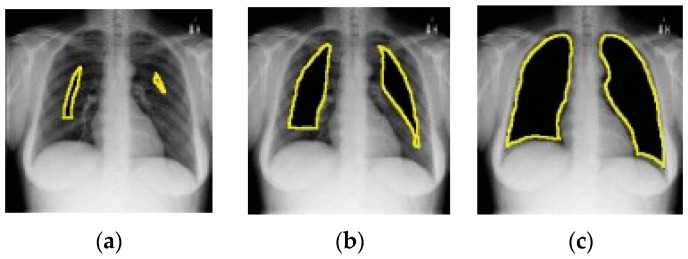
Example of lung occlusion (all images taken from [121]). From (**a**–**c**) the lung area is gradually occluded.

Figure 14 depicts an example of lung occlusion, i.e., the gradual removal of the lung area from CXR images. Ideally, the AUC ROC should go down close to 0.5 for the complete occlusion of the lung. However, an AUC ROC of up to 0.88 has been achieved for full lung occlusion. This is quite remarkable as this means that even without considering lung tissue, the model is able to correctly predict the class of the CXR image. These findings may imply that the results reported in various research studies on the performance of pneumonia detection models may be too optimistic and may not be a true representation of the capabilities of those models.

Recent studies [200,201,202] have proposed ways to mitigate the effects of biased datasets. One possible way [200] is to employ style randomization modules at both image and feature levels to create style-perturbed features while keeping the content intact. This enables CNNs to focus more on the actual, relevant content of CXRs rather than the many different uninformative styles present in biased datasets. Another possibility [201] is to use federated deep learning, ensuring that no single data source can profoundly influence model training. Finally, using ethical tools such as Aequitas [202] to ensure that the dataset is free of biases based on gender, race, age, etc., can help obtain results that are more compatible with ethical and responsible standards.

### 8.2. Data and Code Availability

The majority of the studies surveyed for this paper did not make their code and data public. However, most of the studies in which datasets have been created by combining various publicly available data sources have provided details of the original data sources. But even these studies fail to provide links to their final, combined datasets, except for a few studies like [35,203,204]. Open data and code enable other researchers to validate results [205]. It also helps in the reproduction of the results reported in a study and therefore helps in confirming the truth of results. Moreover, open-source code and data also help in improving existing models and techniques. The reproducibility of pneumonia detection models, therefore, has become a major issue [60]. Current and future research works in this area should ensure open-source code and data availability.

### 8.3. Explainability of Models

The transparency of pneumonia detection models is likely to be critical to their acceptance by medical practitioners [149]. An interpretable and transparent pneumonia detection model can give medical practitioners more confidence in the underlying algorithm and its final prediction [206]. Explainable AI (XAI) can provide a behavioral understanding of these models [119]. The most widely used XAI method for pneumonia detection models is Grad-CAM [71,169,191,207,208,209,210] followed by CAM [133,135]. Other state-of-the-art XAI methods for pneumonia detection models include LIME [211], SHAP [212], and Grad-CAM++ [213]. Recently, new XAI approaches specially designed for CXR-based pneumonia detection models have been presented [119,149].

Up till now, XAI techniques are mostly based on visualizations, i.e., highlighting image areas that contribute to a model’s prediction [210]. These visualizations do help in understanding a model’s behavior, but a more standard, quantitative way of explanation is also required. The creation of standardized quantitative XAI metrics for explainability and interpretability is therefore needed. This can help improve the behavioral understanding of pneumonia detection models in a more standardized and regulated way. There is also a need for a universally agreed definition of ‘explanation’ in the context of XAI [214,215]. In addition, the scope and applicability of XAI should also be clearly defined by some recognized regulatory authority. Future XAI approaches should output explanations in a more comprehensible language that can be interpreted by humans [127].

### 8.4. Fair Comparison

Suppose there are two pneumonia detection models: ‘A’ and ‘B’. Also, suppose there are two separate CXR image datasets: ‘X’ and ‘Y’. Model ‘A’ is evaluated on dataset ‘X’ and model ‘B’ is evaluated on dataset ‘Y’. The performance of model ‘A’ and model ‘B’ cannot be compared because both have been evaluated on different datasets, i.e., ‘X’ and ‘Y’, respectively. It is important to mention that many studies [206] have compared their method(s) evaluated on a particular dataset with other state-of-the-art methods evaluated on different datasets. Such comparisons are not very useful or may even be futile [60]. This is because the performance metrics obtained in each case are data-dependent. There is a need to develop a broad range of labelled, benchmark datasets for the purpose of training and evaluating pneumonia detection models. All models that need to be compared should be evaluated on the same set of datasets. A performance comparison then makes more sense. In this regard, the availability of the code used in published studies can help. This is because this can enable a model to be regenerated and evaluated on benchmark datasets if this has not been already done.

### 8.5. Class Imbalance in CXR Datasets

A key challenge in CXR image classification is having to deal with imbalanced datasets [42]. Class imbalance is very common in COVID-19 CXR image datasets [60] due to unavailability of a large number of COVID-19 samples early on in the pandemic. As a consequence, there are very few COVID-19 samples in many COVID-19 datasets. This results in the algorithm mostly seeing non-COVID-19 samples. An imbalanced dataset, therefore, leads to inaccurate results due to issues such as model overfitting [216,217,218]. This is true for both binary and multi-class classifications.

Deep learning algorithms, such as CNNs, are primarily designed for an equal number of images in each class [219]. If there is a class imbalance, then this needs to be resolved through some technique. Some class imbalance resolution approaches commonly exploited are briefly described here. Rajaraman et al. [220] solved this problem by designing the loss function of their model in a way such that the class with a smaller number of samples is given more weight compared to the class with a greater number of samples. Another mechanism to counter class imbalance is resampling. In [221], resampling is exploited by applying data augmentation to increase the number of samples for the COVID-19 class that had much fewer samples. The objective of resampling is to create an even distribution of classes. This may be accomplished by either increasing the number of samples for the class with fewer samples or by taking fewer samples for the class with a disproportionately higher number of samples. A recent study [222] addresses the issue of class imbalance by exploiting the focal loss (FL) function for imbalanced datasets. The FL function adds a modulating factor that lessens the impact of well-classified examples. The core idea is to concentrate the training process more on difficult, misclassified examples. The FL, therefore, de-emphasizes easy examples to allow the model to focus on the minority class.

### 8.6. Adversarial Attacks

CXR-based pneumonia detection models are highly accurate but are vulnerable to adversarial attacks [223,224,225,226,227,228]. Paschali et al. [229] first suggested that deep learning models in medical imaging should be evaluated for robustness and not just for generalizability. It has been shown in [229] that adversarial examples are a much better means to evaluate a model’s robustness compared to random noise like Gaussian noise. Ma et al. [230] have demonstrated that deep learning models trained and evaluated on medical images such as CXRs are even more vulnerable to adversarial examples compared to models using natural images. This high vulnerability due to adversarial attacks can make automated diagnostic models like CXR-based pneumonia detection models unreliable and a source of fraud and/or confusion.

In [231], a strong, image-independent adversarial attack called a universal adversarial perturbation (UAP) [232] was studied for pneumonia detection. The study [231] exploits a variant of UAP which is based on a single perturbation and which is very easy to apply and computationally a lot less expensive. The results show that the attack has a success rate greater than 80% for both targeted and untargeted attacks. Moreover, adversarial retraining [51], which is a defense mechanism, largely failed to mitigate the effect of the UAP attack [231]. These results highlight the need to focus more on model reliability in case of adversarial attacks in the future.

Some recent studies [233,234] have focused on developing effective defense mechanisms against adversarial attacks on CXR images. Dai et al. [233] employs global attention noise injection to combat adversarial attacks, while Sheikh and Zafar [234] exploit a denoising technique called total variation minimization to effectively mitigate adversarial noise in images. Both these techniques have yielded promising results.

## 9. Conclusions

It is evident from the introductory and explanatory material detailed in sections one to six and the references supplied that a significant body of knowledge and expertise has evolved over time that addresses both related research in the field of and the eventual application of deep learning, especially for pneumonia detection using X-rays. Such work has thus enabled key statistics to be derived in section seven regarding the effectiveness, and hence relative accuracy, of techniques for both detecting and discriminating between forms of COVID and non-COVID pneumonia.

Given the range of both past and present techniques and approaches in deep learning, as well as the evaluations and metrics for effectiveness proposed and detailed herein for its application to pneumonia detection using X-rays, it is possible to show, first, that adversarial attacks are a viable means of identifying, analyzing, and hence determining the effectiveness and reliability (and so ensuring the vital integrity and accuracy) of data models used for the detection of pneumonia via X-rays.

Secondly, it is possible to suggest one particular field of research and its application that may prove particularly useful in such endeavors, i.e., the use of vision transformers (ViTs), though these have inherent limitations that must inevitably be addressed and ultimately resolved.

## Figures and Tables

**Table 1:** Number of search items returned by the research databases.

Electronic Research Database	Search Results (Number of Items)
IEEE Xplore	304
ScienceDirect	160
SpringerLink	240
ACM Digital Library	85

**Table 2:** Some common publicly available CXR image datasets used for COVID and non-COVID pneumonia detection.

Dataset	Link	Studies Using the Dataset	Features		
			No. of Images	No. of Classes	Classes
Kermany’s Dataset [1]	https://data.mendeley.com/datasets/rscbjbr9sj/3 (accessed on 2 February 2024)	[128,129,130,131,132,133,134,135,136,137,138]	5858	3	Viral pneumonia, bacterial pneumonia, normal lungs
RSNA pneumonia dataset [139]	https://www.kaggle.com/c/rsna-pneumonia-detection-challenge (accessed on 3 April 2024)	[140,141,142,143,144,145,146]	26,684	2	Pneumonia and non-pneumonia
NIH Chest X-ray Dataset [147]	https://www.kaggle.com/datasets/nih-chest-xrays/data (accessed on 24 March 2024)	[70,148,149,150,151,152,153,154,155,156]	112,000	15	Atelectasis, consolidation, infiltration, pneumothorax, edema, emphysema, fibrosis, effusion, pneumonia, pleural thickening, cardiomegaly, nodule mass, hernia, no findings
Cohen et al.’s COVID chest X-ray dataset [157,158,159]	https://github.com/ieee8023/covid-chestxray-dataset (accessed on 22 March 2024)	[160,161,162,163]	1314	05	COVID-19 or other viral and bacterial pneumonias (MERS, SARS, and ARDS)
Novel COVID-19 Chestxray Repository [164,165]	https://www.kaggle.com/datasets/subhankarsen/novel-covid19-chestxray-repository (accessed on 14 March 2024)	[165]	3975	3	COVID-19, pneumonia and normal
COVID-19 chest X-ray [166]	https://www.kaggle.com/datasets/ahmedtronic/covid-19-chest-x-ray (accessed on 3 April 2024)	[167]	930	3	COVID-19, pneumonia and normal
Sait et al.’s curated CXR dataset [168]	https://data.mendeley.com/datasets/9xkhgts2s6/4 (accessed on 4 April 2024)	[169,170,171]	9208	4	COVID-19, normal, viral pneumonia and bacterial pneumonia.
Kumar’s COVID-19-Pneumonia-Normal CXR Images dataset [172]	https://data.mendeley.com/datasets/dvntn9yhd2/1 (accessed on 4 April 2024)	[173,174,175,176]	5228	3	COVID-19, pneumonia and normal
Asraf and Islam’s COVID-19, Pneumonia and Normal Chest X-ray PA Dataset [177]	https://data.mendeley.com/datasets/jctsfj2sfn/1 (accessed on 3 April 2024)	[178]	4575	3	COVID-19, pneumonia and normal
COVID-19 Radiography Database [179]	https://www.kaggle.com/datasets/tawsifurrahman/covid19-radiography-database (accessed on 14 March 2024)	[67,160,180,181,182,183]	21,165	4	COVID-19, normal, lung opacity (non-COVID lung infection) and viral pneumonia

**Table 3:** Number of studies for each of the two pneumonia types.

Pneumonia Type	Frequency (Number of Papers)
Non-COVID Pneumonia	22
COVID Pneumonia	48
Both	70

**Table 4:** Examples of binary and multiclass classification tasks.

Binary Classification	Multiclass Classification
“COVID-19 pneumonia” vs. “non-COVID-19 interstitial pneumonia” [8]	“COVID-19 infected pneumonia” vs. “community acquired no COVID-19 infected pneumonia” vs. “normal” [100]
“COVID” vs. “non-COVID” [100]	“COVID” vs. “no findings” vs. “pneumonia” [80]
“COVID” vs. “normal” [79]	“COVID” vs. “normal” vs. “bacterial” vs. “viral” [71,81]
“COVID” vs. “no findings” [80]	“COVID-19” vs. “normal” vs. “viral pneumonia” [91]
“pneumonia” vs. “normal” [83]	“COVID” vs. “normal” vs. “pneumonia” [78]
“bacterial” vs. “viral” [184]	“COVID-19” vs. “pneumonia” vs. “pneumothorax” vs. “tuberculosis” vs. “normal” [47]

## References

[1] Kermany D.S., Goldbaum M., Cai W., Valentim C.C.S., Liang H., Baxter S.L., McKeown A., Yang G., Wu X., Yan F. (2018). Identifying Medical Diagnoses and Treatable Diseases by Image-Based Deep Learning. Cell.

[2] Ibrahim A.U., Ozsoz M., Serte S., Al-Turjman F., Yakoi P.S. (2024). Pneumonia Classification Using Deep Learning from Chest X-ray Images During COVID-19. Cogn. Comput..

[3] Pneumonia|CDC. https://www.cdc.gov/pneumonia/index.html.

[4] Ruuskanen O., Lahti E., Jennings L.C., Murdoch D.R. (2011). Viral pneumonia. Lancet.

[5] World Health Organization (WHO). https://www.who.int/.

[6] Khan W., Zaki N., Ali L. (2021). Intelligent Pneumonia Identification From Chest X-Rays: A Systematic Literature Review. IEEE Access.

[7] Johns Hopkins Coronavirus Resource Center Johns Hopkins University & Medicine. https://coronavirus.jhu.edu/.

[8] Ieracitano C., Mammone N., Versaci M., Varone G., Ali A.-R., Armentano A., Calabrese G., Ferrarelli A., Turano L., Tebala C. (2022). A fuzzy-enhanced deep learning approach for early detection of COVID-19 pneumonia from portable chest X-ray images. Neurocomputing.

[9] D’Arienzo M., Coniglio A. (2020). Assessment of the SARS-CoV-2 basic reproduction number, R0, based on the early phase of COVID-19 outbreak in Italy. Biosaf. Health.

[10] Rossman H., Meir T., Somer J., Shilo S., Gutman R., Ben Arie A., Segal E., Shalit U., Gorfine M. (2021). Hospital load and increased COVID-19 related mortality in Israel. Nat. Commun..

[11] Yaseliani M., Hamadani A.Z., Maghsoodi A.I., Mosavi A. (2022). Pneumonia Detection Proposing a Hybrid Deep Convolutional Neural Network Based on Two Parallel Visual Geometry Group Architectures and Machine Learning Classifiers. IEEE Access.

[12] Self W.H., Courtney D.M., McNaughton C.D., Wunderink R.G., Kline J.A. (2013). High discordance of chest X-ray and computed tomography for detection of pulmonary opacities in ED patients: Implications for diagnosing pneumonia. Am. J. Emerg. Med..

[13] Ticinesi A., Lauretani F., Nouvenne A., Mori G., Chiussi G., Maggio M., Meschi T. (2016). Lung ultrasound and chest X-ray for detecting pneumonia in an acute geriatric ward. Medicine.

[14] Htun T.P., Sun Y., Chua H.L., Pang J. (2019). Clinical features for diagnosis of pneumonia among adults in primary care setting: A systematic and meta-review. Sci. Rep..

[15] Siddiqi R. (2020). Efficient Pediatric Pneumonia Diagnosis Using Depthwise Separable Convolutions. SN Comput. Sci..

[16] Mettler F.A., Huda W., Yoshizumi T.T., Mahesh M. (2008). Effective Doses in Radiology and Diagnostic Nuclear Medicine: A Catalog. Radiology.

[17] Çallı E., Sogancioglu E., van Ginneken B., van Leeuwen K.G., Murphy K. (2021). Deep learning for chest X-ray analysis: A survey. Med. Image Anal..

[18] Wang L., Lin Z.Q., Wong A. (2020). COVID-Net: A tailored deep convolutional neural network design for detection of COVID-19 cases from chest X-ray images. Sci. Rep..

[19] Ai T., Yang Z., Hou H., Zhan C., Chen C., Lv W., Tao Q., Sun Z., Xia L. (2020). Correlation of Chest CT and RT-PCR Testing for Coronavirus Disease 2019 (COVID-19) in China: A Report of 1014 Cases. Radiology.

[20] Fang Y., Zhang H., Xie J., Lin M., Ying L., Pang P., Ji W. (2020). Sensitivity of Chest CT for COVID-19: Comparison to RT-PCR. Radiology.

[21] Jacobi A., Chung M., Bernheim A., Eber C. (2020). Portable chest X-ray in coronavirus disease-19 (COVID-19): A pictorial review. Clin. Imaging.

[22] Pal B., Gupta D., Rashed-Al-Mahfuz M., Alyami S.A., Moni M.A. (2021). Vulnerability in Deep Transfer Learning Models to Adversarial Fast Gradient Sign Attack for COVID-19 Prediction from Chest Radiography Images. Appl. Sci..

[23] Rajaraman S., Guo P., Xue Z., Antani S.K. (2022). A Deep Modality-Specific Ensemble for Improving Pneumonia Detection in Chest X-rays. Diagnostics.

[24] Kundu R., Das R., Geem Z.W., Han G.-T., Sarkar R. (2021). Pneumonia detection in chest X-ray images using an ensemble of deep learning models. PLoS ONE.

[25] Mousavi Z., Shahini N., Sheykhivand S., Mojtahedi S., Arshadi A. (2022). COVID-19 detection using chest X-ray images based on a developed deep neural network. SLAS Technol..

[26] Cha S.-M., Lee S.-S., Ko B. (2021). Attention-Based Transfer Learning for Efficient Pneumonia Detection in Chest X-ray Images. Appl. Sci..

[27] LeCun Y., Bengio Y., Hinton G. (2015). Deep learning. Nature.

[28] Sarker I.H. (2021). Deep Learning: A Comprehensive Overview on Techniques, Taxonomy, Applications and Research Directions. SN Comput. Sci..

[29] Zhou S.K., Greenspan H., Davatzikos C., Duncan J.S., Van Ginneken B., Madabhushi A., Prince J.L., Rueckert D., Summers R.M. (2021). A Review of Deep Learning in Medical Imaging: Imaging Traits, Technology Trends, Case Studies With Progress Highlights, and Future Promises. Proc. IEEE.

[30] Yamashita R., Nishio M., Do R.K.G., Togashi K. (2018). Convolutional neural networks: An overview and application in radiology. Insights Imaging.

[31] Irvin J., Rajpurkar P., Ko M., Yu Y., Ciurea-Ilcus S., Chute C., Marklund H., Haghgoo B., Ball R., Shpanskaya K. (2019). CheXpert: A Large Chest Radiograph Dataset with Uncertainty Labels and Expert Comparison. Proc. AAAI Conf. Artif. Intell..

[32] Wang X., Peng Y., Lu L., Lu Z., Bagheri M., Summers R.M. ChestX-Ray8: Hospital-Scale Chest X-Ray Database and Benchmarks on Weakly-Supervised Classification and Localization of Common Thorax Diseases. Proceedings of the 2017 IEEE Conference on Computer Vision and Pattern Recognition (CVPR).

[33] Johnson A.E.W., Pollard T.J., Berkowitz S.J., Greenbaum N.R., Lungren M.P., Deng C., Mark R.G., Horng S. (2019). MIMIC-CXR, a de-identified publicly available database of chest radiographs with free-text reports. Sci. Data.

[34] Bustos A., Pertusa A., Salinas J.-M., de la Iglesia-Vayá M. (2020). PadChest: A large chest X-ray image dataset with multi-label annotated reports. Med. Image Anal..

[35] Tabik S., Gómez-Ríos A., Martín-Rodríguez J.L., Sevillano-García I., Rey-Area M., Charte D., Guirado E., Suárez J.L., Luengo J., Valero-González M.A. (2020). COVIDGR Dataset and COVID-SDNet Methodology for Predicting COVID-19 Based on Chest X-Ray Images. IEEE J. Biomed. Health Inform..

[36] Srivastav D., Bajpai A., Srivastava P. Improved Classification for Pneumonia Detection using Transfer Learning with GAN based Synthetic Image Augmentation. Proceedings of the 2021 11th International Conference on Cloud Computing, Data Science & Engineering.

[37] Motamed S., Rogalla P., Khalvati F. (2021). Data augmentation using Generative Adversarial Networks (GANs) for GAN-based detection of Pneumonia and COVID-19 in chest X-ray images. Inform. Med. Unlocked.

[38] Russakovsky O., Deng J., Su H., Krause J., Satheesh S., Ma S., Huang Z., Karpathy A., Khosla A., Bernstein M. (2015). ImageNet Large Scale Visual Recognition Challenge. Int. J. Comput. Vis..

[39] Sousa R.T., Marques O., Soares F.A.A.M.N., Sene I.I.G., de Oliveira L.L.G., Spoto E.S. (2013). Comparative Performance Analysis of Machine Learning Classifiers in Detection of Childhood Pneumonia Using Chest Radiographs. Procedia Comput. Sci..

[40] Khobragade S., Tiwari A., Patil C.Y., Narke V. Automatic detection of major lung diseases using Chest Radiographs and classification by feed-forward artificial neural network. Proceedings of the 2016 IEEE 1st International Conference on Power Electronics, Intelligent Control and Energy Systems (ICPEICES).

[41] Hussain K., Mohd Salleh M.N., Cheng S., Shi Y. (2019). Metaheuristic research: A comprehensive survey. Artif. Intell. Rev..

[42] Meedeniya D., Kumarasinghe H., Kolonne S., Fernando C., Díez I.D.l.T., Marques G. (2022). Chest X-ray analysis empowered with deep learning: A systematic review. Appl. Soft Comput..

[43] Alghamdi H.S., Amoudi G., Elhag S., Saeedi K., Nasser J. (2021). Deep Learning Approaches for Detecting COVID-19 From Chest X-Ray Images: A Survey. IEEE Access.

[44] Shorten C., Khoshgoftaar T.M. (2019). A survey on Image Data Augmentation for Deep Learning. J. Big Data.

[45] Ahmed K.B., Goldgof G.M., Paul R., Goldgof D.B., Hall L.O. (2021). Discovery of a Generalization Gap of Convolutional Neural Networks on COVID-19 X-Rays Classification. IEEE Access.

[46] El-Kenawy E.-S.M., Mirjalili S., Ibrahim A., Alrahmawy M., El-Said M., Zaki R.M., Eid M.M. (2021). Advanced Meta-Heuristics, Convolutional Neural Networks, and Feature Selectors for Efficient COVID-19 X-Ray Chest Image Classification. IEEE Access.

[47] Karaddi S.H., Sharma L.D. (2023). Automated multi-class classification of lung diseases from CXR-images using pre-trained convolutional neural networks. Expert Syst. Appl..

[48] Wong S.C., Gatt A., Stamatescu V., McDonnell M.D. Understanding Data Augmentation for Classification: When to Warp?. Proceedings of the 2016 International Conference on Digital Image Computing: Techniques and Applications (DICTA).

[49] Wang Q., Zhou X., Wang C., Liu Z., Huang J., Zhou Y., Li C., Zhuang H., Cheng J.-Z. (2019). WGAN-Based Synthetic Minority Over-Sampling Technique: Improving Semantic Fine-Grained Classification for Lung Nodules in CT Images. IEEE Access.

[50] Zhong Z., Zheng L., Kang G., Li S., Yang Y. (2020). Random Erasing Data Augmentation. Proc. AAAI Conf. Artif. Intell..

[51] Siddiqi R. (2021). Fruit-classification model resilience under adversarial attack. SN Appl. Sci..

[52] Chlap P., Min H., Vandenberg N., Dowling J., Holloway L., Haworth A. (2021). A review of medical image data augmentation techniques for deep learning applications. J. Med. Imaging Radiat. Oncol..

[53] Wang K., Gou C., Duan Y., Lin Y., Zheng X., Wang F.-Y. (2017). Generative adversarial networks: Introduction and outlook. IEEECAA J. Autom. Sin..

[54] Gui J., Sun Z., Wen Y., Tao D., Ye J. (2021). A Review on Generative Adversarial Networks: Algorithms, Theory, and Applications. IEEE Trans. Knowl. Data Eng..

[55] Gulakala R., Markert B., Stoffel M. (2023). Rapid diagnosis of Covid-19 infections by a progressively growing GAN and CNN optimisation. Comput. Methods Programs Biomed..

[56] Gulakala R., Markert B., Stoffel M. (2022). Generative adversarial network based data augmentation for CNN based detection of COVID-19. Sci. Rep..

[57] Khalifa N.E.M., Taha M.H.N., Hassanien A.E., Elghamrawy S., Hassanien A.E., Snášel V., Tang M., Sung T.-W., Chang K.-C. (2023). Detection of Coronavirus (COVID-19) Associated Pneumonia Based on Generative Adversarial Networks and a Fine-Tuned Deep Transfer Learning Model Using Chest X-ray Dataset. Proceedings of the 8th International Conference on Advanced Intelligent Systems and Informatics 2022.

[58] Loey M., Smarandache F., Khalifa N.E.M. (2020). Within the Lack of Chest COVID-19 X-ray Dataset: A Novel Detection Model Based on GAN and Deep Transfer Learning. Symmetry.

[59] Jabbar A., Li X., Omar B. (2022). A Survey on Generative Adversarial Networks: Variants, Applications, and Training. ACM Comput. Surv..

[60] Aggarwal P., Mishra N.K., Fatimah B., Singh P., Gupta A., Joshi S.D. (2022). COVID-19 image classification using deep learning: Advances, challenges and opportunities. Comput. Biol. Med..

[61] Tan Y., Tan Y. (2016). Chapter 11—Applications. Gpu-Based Parallel Implementation of Swarm Intelligence Algorithms.

[62] Narayanan B.N., Davuluru V.S.P., Hardie R.C. (2020). Two-stage deep learning architecture for pneumonia detection and its diagnosis in chest radiographs. Proceedings of the Medical Imaging 2020: Imaging Informatics for Healthcare, Research, and Applications.

[63] Ronneberger O., Fischer P., Brox T., Navab N., Hornegger J., Wells W.M., Frangi A.F. (2015). U-Net: Convolutional Networks for Biomedical Image Segmentation. Proceedings of the Medical Image Computing and Computer-Assisted Intervention–MICCAI 2015.

[64] Long J., Shelhamer E., Darrell T. Fully Convolutional Networks for Semantic Segmentation. Proceedings of the IEEE Conference on Computer Vision and Pattern Recognition 2015.

[65] Milletari F., Navab N., Ahmadi S.-A. V-Net: Fully Convolutional Neural Networks for Volumetric Medical Image Segmentation. Proceedings of the 2016 Fourth International Conference on 3D Vision (3DV).

[66] Chen J., Cai Z., Heidari A.A., Liu L., Chen H., Pan J. (2023). Dynamic mechanism-assisted artificial bee colony optimization for image segmentation of COVID-19 chest X-ray. Displays.

[67] Al-Zyoud W., Erekat D., Saraiji R. (2023). COVID-19 chest X-ray image analysis by threshold-based segmentation. Heliyon.

[68] Rahman M.F., Zhuang Y., Tseng T.-L.B., Pokojovy M., McCaffrey P., Walser E., Moen S., Vo A. (2022). Improving lung region segmentation accuracy in chest X-ray images using a two-model deep learning ensemble approach. J. Vis. Commun. Image Represent..

[69] Manickam A., Jiang J., Zhou Y., Sagar A., Soundrapandiyan R., Dinesh Jackson Samuel R. (2021). Automated pneumonia detection on chest X-ray images: A deep learning approach with different optimizers and transfer learning architectures. Measurement.

[70] Ortiz-Toro C., García-Pedrero A., Lillo-Saavedra M., Gonzalo-Martín C. (2022). Automatic detection of pneumonia in chest X-ray images using textural features. Comput. Biol. Med..

[71] Karthik R., Menaka R., Hariharan M. (2021). Learning distinctive filters for COVID-19 detection from chest X-ray using shuffled residual CNN. Appl. Soft Comput..

[72] Chollet F. (2021). Deep Learning with Python.

[73] Khan M., Mehran M.T., Haq Z.U., Ullah Z., Naqvi S.R., Ihsan M., Abbass H. (2021). Applications of artificial intelligence in COVID-19 pandemic: A comprehensive review. Expert Syst. Appl..

[74] Babukarthik R.G., Adiga V.A.K., Sambasivam G., Chandramohan D., Amudhavel J. (2020). Prediction of COVID-19 Using Genetic Deep Learning Convolutional Neural Network (GDCNN). IEEE Access.

[75] Singh G., Yow K.-C. (2021). An Interpretable Deep Learning Model for COVID-19 Detection With Chest X-Ray Images. IEEE Access.

[76] Rozenberg E., Freedman D., Bronstein A.A. (2021). Learning to Localize Objects Using Limited Annotation, with Applications to Thoracic Diseases. IEEE Access.

[77] (2021). Advance Warning Methodologies for COVID-19 Using Chest X-ray Images. IEEE Access.

[78] Singh G., Yow K.-C. (2021). These do not Look Like Those: An Interpretable Deep Learning Model for Image Recognition. IEEE Access.

[79] Ismael A.M., Şengür A. (2021). Deep learning approaches for COVID-19 detection based on chest X-ray images. Expert Syst. Appl..

[80] Ozturk T., Talo M., Yildirim E.A., Baloglu U.B., Yildirim O., Rajendra Acharya U. (2020). Automated detection of COVID-19 cases using deep neural networks with X-ray images. Comput. Biol. Med..

[81] Reis H.C., Turk V. (2022). COVID-DSNet: A novel deep convolutional neural network for detection of coronavirus (SARS-CoV-2) cases from CT and Chest X-Ray images. Artif. Intell. Med..

[82] Szepesi P., Szilágyi L. (2022). Detection of pneumonia using convolutional neural networks and deep learning. Biocybern. Biomed. Eng..

[83] Kusk M.W., Lysdahlgaard S. (2023). The effect of Gaussian noise on pneumonia detection on chest radiographs, using convolutional neural networks. Radiography.

[84] Umer M., Ashraf I., Ullah S., Mehmood A., Choi G.S. (2022). COVINet: A convolutional neural network approach for predicting COVID-19 from chest X-ray images. J. Ambient Intell. Humaniz. Comput..

[85] Rajawat N., Hada B.S., Meghawat M., Lalwani S., Kumar R. (2022). C-COVIDNet: A CNN Model for COVID-19 Detection Using Image Processing. Arab. J. Sci. Eng..

[86] Sedik A., Hammad M., Abd El-Samie F.E., Gupta B.B., Abd El-Latif A.A. (2022). Efficient deep learning approach for augmented detection of Coronavirus disease. Neural Comput. Appl..

[87] Subramanian N., Elharrouss O., Al-Maadeed S., Chowdhury M. (2022). A review of deep learning-based detection methods for COVID-19. Comput. Biol. Med..

[88] Avola D., Bacciu A., Cinque L., Fagioli A., Marini M.R., Taiello R. (2022). Study on transfer learning capabilities for pneumonia classification in chest-x-rays images. Comput. Methods Programs Biomed..

[89] Katsamenis I., Protopapadakis E., Voulodimos A., Doulamis A., Doulamis N. (2021). Transfer Learning for COVID-19 Pneumonia Detection and Classification in Chest X-ray Images. Proceedings of the 24th Pan-Hellenic Conference on Informatics.

[90] Pan S.J., Yang Q. (2010). A Survey on Transfer Learning. IEEE Trans. Knowl. Data Eng..

[91] Chamseddine E., Mansouri N., Soui M., Abed M. (2022). Handling class imbalance in COVID-19 chest X-ray images classification: Using SMOTE and weighted loss. Appl. Soft Comput..

[92] Ahsan M.M., Ahad M.T., Soma F.A., Paul S., Chowdhury A., Luna S.A., Yazdan M.S., Rahman A., Siddique Z., Huebner P. (2021). Detecting SARS-CoV-2 From Chest X-ray Using Artificial Intelligence. IEEE Access.

[93] Aslan M.F., Unlersen M.F., Sabanci K., Durdu A. (2021). CNN-based transfer learning–BiLSTM network: A novel approach for COVID-19 infection detection. Appl. Soft Comput..

[94] Karacı A. (2022). VGGCOV19-NET: Automatic detection of COVID-19 cases from X-ray images using modified VGG19 CNN architecture and YOLO algorithm. Neural Comput. Appl..

[95] Tahir A.M., Qiblawey Y., Khandakar A., Rahman T., Khurshid U., Musharavati F., Islam M.T., Kiranyaz S., Al-Maadeed S., Chowdhury M.E.H. (2022). Deep Learning for Reliable Classification of COVID-19, MERS, and SARS from Chest X-ray Images. Cogn. Comput..

[96] Chouat I., Echtioui A., Khemakhem R., Zouch W., Ghorbel M., Hamida A.B. (2022). COVID-19 detection in CT and CXR images using deep learning models. Biogerontology.

[97] Dialameh M., Hamzeh A., Rahmani H., Radmard A.R., Dialameh S. (2022). Proposing a novel deep network for detecting COVID-19 based on chest images. Sci. Rep..

[98] Shoaib M.R., Emara H.M., Elwekeil M., El-Shafai W., Taha T.E., El-Fishawy A.S., El-Rabaie E.-S.M., El-Samie F.E.A. (2022). Hybrid classification structures for automatic COVID-19 detection. J. Ambient Intell. Humaniz. Comput..

[99] Sundaram S.G., Aloyuni S.A., Alharbi R.A., Alqahtani T., Sikkandar M.Y., Subbiah C. (2022). Deep Transfer Learning Based Unified Framework for COVID19 Classification and Infection Detection from Chest X-Ray Images. Arab. J. Sci. Eng..

[100] Heidari M., Mirniaharikandehei S., Khuzani A.Z., Danala G., Qiu Y., Zheng B. (2020). Improving the performance of CNN to predict the likelihood of COVID-19 using chest X-ray images with preprocessing algorithms. Int. J. Med. Inf..

[101] Imagawa K., Shiomoto K. (2022). Performance change with the number of training data: A case study on the binary classification of COVID-19 chest X-ray by using convolutional neural networks. Comput. Biol. Med..

[102] Gazda M., Plavka J., Gazda J., Drotár P. (2021). Self-Supervised Deep Convolutional Neural Network for Chest X-Ray Classification. IEEE Access.

[103] Jena B., Saxena S., Nayak G.K., Saba L., Sharma N., Suri J.S. (2021). Artificial intelligence-based hybrid deep learning models for image classification: The first narrative review. Comput. Biol. Med..

[104] Shouman M.A., El-Fiky A., Hamada S., El-Sayed A., Karar M.E. (2022). Computer-assisted lung diseases detection from pediatric chest radiography using long short-term memory networks. Comput. Electr. Eng..

[105] Sourab S.Y., Kabir M.A. (2022). A comparison of hybrid deep learning models for pneumonia diagnosis from chest radiograms. Sens. Int..

[106] Jin W., Dong S., Dong C., Ye X. (2021). Hybrid ensemble model for differential diagnosis between COVID-19 and common viral pneumonia by chest X-ray radiograph. Comput. Biol. Med..

[107] Nandi R., Mulimani M. (2021). Detection of COVID-19 from X-rays using hybrid deep learning models. Res. Biomed. Eng..

[108] Shah P.M., Ullah F., Shah D., Gani A., Maple C., Wang Y., Shahid, Abrar M., Islam S.U. (2022). Deep GRU-CNN Model for COVID-19 Detection From Chest X-rays Data. IEEE Access.

[109] Kaya M. (2024). Feature fusion-based ensemble CNN learning optimization for automated detection of pediatric pneumonia. Biomed. Signal Process. Control.

[110] Bhatt H., Shah M. (2023). A Convolutional Neural Network ensemble model for Pneumonia Detection using chest X-ray images. Healthc. Anal..

[111] Srivastava G., Pradhan N., Saini Y. (2022). Ensemble of Deep Neural Networks based on Condorcet’s Jury Theorem for screening COVID-19 and Pneumonia from radiograph images. Comput. Biol. Med..

[112] Mabrouk A., Díaz Redondo R.P., Abd Elaziz M., Kayed M. (2023). Ensemble Federated Learning: An approach for collaborative pneumonia diagnosis. Appl. Soft Comput..

[113] Sahoo P., Saha S., Sharma S.K., Mondal S., Gowda S. (2023). A Multi-stage framework for COVID-19 detection and severity assessment from chest radiography images using advanced fuzzy ensemble technique. Expert Syst. Appl..

[114] Balasubramaniam S., Satheesh Kumar K. (2023). Optimal Ensemble learning model for COVID-19 detection using chest X-ray images. Biomed. Signal Process. Control.

[115] Kushal K.S., Ahmed T., Uddin M.A., Uddin M.N. (2023). A Blockchain-Based Framework for COVID-19 Detection Using Stacking Ensemble of Pre-Trained Models. Comput. Methods Programs Biomed. Update.

[116] Zhang Y., Weng Y., Lund J. (2022). Applications of Explainable Artificial Intelligence in Diagnosis and Surgery. Diagnostics.

[117] Tjoa E., Guan C. (2021). A Survey on Explainable Artificial Intelligence (XAI): Toward Medical XAI. IEEE Trans. Neural Netw. Learn. Syst..

[118] Adadi A., Berrada M. (2018). Peeking Inside the Black-Box: A Survey on Explainable Artificial Intelligence (XAI). IEEE Access.

[119] Zou L., Goh H.L., Liew C.J.Y., Quah J.L., Gu G.T., Chew J.J., Prem Kumar M., Ang C.G.L., Ta A. (2022). Ensemble image explainable AI (XAI) algorithm for severe community-acquired pneumonia and COVID-19 respiratory infections. IEEE Trans. Artif. Intell..

[120] Ren H., Wong A.B., Lian W., Cheng W., Zhang Y., He J., Liu Q., Yang J., Zhang C.J., Wu K. (2021). Interpretable Pneumonia Detection by Combining Deep Learning and Explainable Models With Multisource Data. IEEE Access.

[121] Catalá O.D.T., Igual I.S., Pérez-Benito F.J., Escrivá D.M., Castelló V.O., Llobet R., Peréz-Cortés J.-C. (2021). Bias Analysis on Public X-Ray Image Datasets of Pneumonia and COVID-19 Patients. IEEE Access.

[122] Minaee S., Kafieh R., Sonka M., Yazdani S., Jamalipour Soufi G. (2020). Deep-COVID: Predicting COVID-19 from chest X-ray images using deep transfer learning. Med. Image Anal..

[123] Marques G., Agarwal D., de la Torre Díez I. (2020). Automated medical diagnosis of COVID-19 through EfficientNet convolutional neural network. Appl. Soft Comput..

[124] Oh Y., Park S., Ye J.C. (2020). Deep Learning COVID-19 Features on CXR Using Limited Training Data Sets. IEEE Trans. Med. Imaging.

[125] Das D., Santosh K.C., Pal U. (2020). Truncated inception net: COVID-19 outbreak screening using chest X-rays. Phys. Eng. Sci. Med..

[126] Mukherjee H., Ghosh S., Dhar A., Obaidullah S.M., Santosh K.C., Roy K. (2021). Shallow Convolutional Neural Network for COVID-19 Outbreak Screening Using Chest X-rays. Cogn. Comput..

[127] Ghassemi M., Oakden-Rayner L., Beam A.L. (2021). The false hope of current approaches to explainable artificial intelligence in health care. Lancet Digit. Health.

[128] Bodapati J.D., Rohith V.N., Dondeti V. (2022). Ensemble of deep capsule neural networks: An application to pediatric pneumonia prediction. Phys. Eng. Sci. Med..

[129] Bodapati J.D., Rohith V.N. (2022). ChxCapsNet: Deep capsule network with transfer learning for evaluating pneumonia in paediatric chest radiographs. Measurement.

[130] Kör H., Erbay H., Yurttakal A.H. (2022). Diagnosing and differentiating viral pneumonia and COVID-19 using X-ray images. Multimed. Tools Appl..

[131] Sasikaladevi N., Revathi A. (2023). Intelligent prognostic system for pediatric pneumonia based on sustainable IoHT. Multimed. Tools Appl..

[132] Liz H., Sánchez-Montañés M., Tagarro A., Domínguez-Rodríguez S., Dagan R., Camacho D. (2021). Ensembles of Convolutional Neural Network models for pediatric pneumonia diagnosis. Future Gener. Comput. Syst..

[133] Prakash J.A., Ravi V., Sowmya V., Soman K.P. (2023). Stacked ensemble learning based on deep convolutional neural networks for pediatric pneumonia diagnosis using chest X-ray images. Neural Comput. Appl..

[134] Arun Prakash J., Asswin C.R., Dharshan Kumar K.S., Avinash D., Vinayakumar R., Sowmya V., Gopalakrishnan E.A., Soman K.P. (2023). Transfer learning approach for pediatric pneumonia diagnosis using channel attention deep CNN architectures. Eng. Appl. Artif. Intell..

[135] Arun Prakash J., Asswin C., Ravi V., Sowmya V., Soman K. (2023). Pediatric pneumonia diagnosis using stacked ensemble learning on multi-model deep CNN architectures. Multimed. Tools Appl..

[136] Gaur L., Bhatia U., Jhanjhi N.Z., Muhammad G., Masud M. (2023). Medical image-based detection of COVID-19 using Deep Convolution Neural Networks. Multimed. Syst..

[137] Paul A., Basu A., Mahmud M., Kaiser M.S., Sarkar R. (2023). Inverted bell-curve-based ensemble of deep learning models for detection of COVID-19 from chest X-rays. Neural Comput. Appl..

[138] Mondal A.K. (2022). COVID-19 prognosis using limited chest X-ray images. Appl. Soft Comput..

[139] RSNA Pneumonia Detection Challenge. https://kaggle.com/competitions/rsna-pneumonia-detection-challenge.

[140] Lakhani P., Mongan J., Singhal C., Zhou Q., Andriole K.P., Auffermann W.F., Prasanna P.M., Pham T.X., Peterson M., Bergquist P.J. (2023). The 2021 SIIM-FISABIO-RSNA Machine Learning COVID-19 Challenge: Annotation and Standard Exam Classification of COVID-19 Chest Radiographs. J. Digit. Imaging.

[141] Fu Y., Xue P., Zhang Z., Dong E. (2023). PKA2-Net: Prior Knowledge-Based Active Attention Network for Accurate Pneumonia Diagnosis on Chest X-ray Images. IEEE J. Biomed. Health Inform..

[142] Feng Y., Wang Z., Xu X., Wang Y., Fu H., Li S., Zhen L., Lei X., Cui Y., Sim Zheng Ting J. (2023). Contrastive domain adaptation with consistency match for automated pneumonia diagnosis. Med. Image Anal..

[143] Malik H., Anees T., Din M., Naeem A. (2023). CDC_Net: Multi-classification convolutional neural network model for detection of COVID-19, pneumothorax, pneumonia, lung Cancer, and tuberculosis using chest X-rays. Multimed. Tools Appl..

[144] Ahmad M., Bajwa U.I., Mehmood Y., Anwar M.W. (2023). Lightweight ResGRU: A deep learning-based prediction of SARS-CoV-2 (COVID-19) and its severity classification using multimodal chest radiography images. Neural Comput. Appl..

[145] Elhanashi A., Saponara S., Zheng Q. (2023). Classification and Localization of Multi-Type Abnormalities on Chest X-rays Images. IEEE Access.

[146] Khero K., Usman M., Fong A. (2023). Deep learning framework for early detection of COVID-19 using X-ray images. Multimed. Tools Appl..

[147] NIH Chest X-rays. https://www.kaggle.com/datasets/nih-chest-xrays/data.

[148] Santomartino S.M., Hafezi-Nejad N., Parekh V.S., Yi P.H. (2023). Performance and Usability of Code-Free Deep Learning for Chest Radiograph Classification, Object Detection, and Segmentation. Radiol. Artif. Intell..

[149] Sheu R.-K., Pardeshi M.S., Pai K.-C., Chen L.-C., Wu C.-L., Chen W.-C. (2023). Interpretable Classification of Pneumonia Infection Using eXplainable AI (XAI-ICP). IEEE Access.

[150] Chetoui M., Akhloufi M.A., Bouattane E.M., Abdulnour J., Roux S., Bernard C.D. (2023). Explainable COVID-19 Detection Based on Chest X-rays Using an End-to-End RegNet Architecture. Viruses.

[151] Li F., Lu X., Yuan J. (2022). MHA-CoroCapsule: Multi-Head Attention Routing-Based Capsule Network for COVID-19 Chest X-Ray Image Classification. IEEE Trans. Med. Imaging.

[152] de Moura J., Novo J., Ortega M. (2022). Fully automatic deep convolutional approaches for the analysis of COVID-19 using chest X-ray images. Appl. Soft Comput..

[153] Nahiduzzaman, Goni O.F., Hassan R., Islam R., Syfullah M.K., Shahriar S.M., Anower S., Ahsan M., Haider J., Kowalski M. (2023). Parallel CNN-ELM: A multiclass classification of chest X-ray images to identify seventeen lung diseases including COVID-19. Expert Syst. Appl..

[154] Rajagopal R., Karthick R., Meenalochini P., Kalaichelvi T. (2023). Deep Convolutional Spiking Neural Network optimized with Arithmetic optimization algorithm for lung disease detection using chest X-ray images. Biomed. Signal Process. Control.

[155] Bhosale Y.H., Patnaik K.S. (2023). PulDi-COVID: Chronic obstructive pulmonary (lung) diseases with COVID-19 classification using ensemble deep convolutional neural network from chest X-ray images to minimize severity and mortality rates. Biomed. Signal Process. Control.

[156] Xin K.Z., Li D., Yi P.H. (2022). Limited generalizability of deep learning algorithm for pediatric pneumonia classification on external data. Emerg. Radiol..

[157] Cohen J.P. Ieee8023/Covid-Chestxray-Dataset. https://github.com/ieee8023/covid-chestxray-dataset.

[158] Cohen J.P., Morrison P., Dao L. (2020). COVID-19 Image Data Collection. arxiv.

[159] Cohen J.P., Morrison P., Dao L., Roth K., Duong T.Q., Ghassemi M. (2020). COVID-19 Image Data Collection: Prospective Predictions Are the Future. Mach. Learn. Biomed. Imaging.

[160] Pham T.D. (2020). Classification of COVID-19 chest X-rays with deep learning: New models or fine tuning?. Health Inf. Sci. Syst..

[161] Patro K.K., Allam J.P., Hammad M., Tadeusiewicz R., Pławiak P. (2023). SCovNet: A skip connection-based feature union deep learning technique with statistical approach analysis for the detection of COVID-19. Biocybern. Biomed. Eng..

[162] Raghavendran P.S., Ragul S., Asokan R., Loganathan A.K., Muthusamy S., Mishra O.P., Ramamoorthi P., Sundararajan S.C.M. (2023). A new method for chest X-ray images categorization using transfer learning and CovidNet_2020 employing convolution neural network. Soft Comput..

[163] Wang T., Nie Z., Wang R., Xu Q., Huang H., Xu H., Xie F., Liu X.-J. (2023). PneuNet: Deep learning for COVID-19 pneumonia diagnosis on chest X-ray image analysis using Vision Transformer. Med. Biol. Eng. Comput..

[164] Novel COVID-19 Chestxray Repository. https://www.kaggle.com/datasets/subhankarsen/novel-covid19-chestxray-repository.

[165] Bhowal P., Sen S., Yoon J.H., Geem Z.W., Sarkar R. (2021). Choquet Integral and Coalition Game-Based Ensemble of Deep Learning Models for COVID-19 Screening From Chest X-ray Images. IEEE J. Biomed. Health Inform..

[166] COVID-19 Chest X-ray. https://www.kaggle.com/datasets/ahmedtronic/covid-19-chest-x-ray.

[167] Haghanifar A., Majdabadi M.M., Choi Y., Deivalakshmi S., Ko S. (2022). COVID-CXNet: Detecting COVID-19 in frontal chest X-ray images using deep learning. Multimed. Tools Appl..

[168] Sait U., Lal KV G., Prakash Prajapati S., Bhaumik R., Kumar T., Shivakumar S., Bhalla K. Curated Dataset for COVID-19 Posterior-Anterior Chest Radiography Images (X-rays). 2021, Version 3. https://data.mendeley.com/datasets/9xkhgts2s6/3.

[169] Mahmud T., Rahman M.A., Fattah S.A. (2020). CovXNet: A multi-dilation convolutional neural network for automatic COVID-19 and other pneumonia detection from chest X-ray images with transferable multi-receptive feature optimization. Comput. Biol. Med..

[170] Sait U., Lal K.V. G., Shivakumar S., Kumar T., Bhaumik R., Prajapati S., Bhalla K., Chakrapani A. (2021). A deep-learning based multimodal system for COVID-19 diagnosis using breathing sounds and chest X-ray images. Appl. Soft Comput..

[171] Hariri M., Avşar E. (2023). COVID-19 and pneumonia diagnosis from chest X-ray images using convolutional neural networks. Netw. Model. Anal. Health Inform. Bioinform..

[172] Kumar S. COVID-19-Pneumonia-Normal Chest X-Ray Images. 2022, Version 1. https://www.sciencedirect.com/science/article/pii/S1568494621004452?via%3Dihub.

[173] Shastri S., Kansal I., Kumar S., Singh K., Popli R., Mansotra V. (2022). CheXImageNet: A novel architecture for accurate classification of COVID-19 with chest X-ray digital images using deep convolutional neural networks. Health Technol..

[174] Kumar S., Shastri S., Mahajan S., Singh K., Gupta S., Rani R., Mohan N., Mansotra V. (2022). LiteCovidNet: A lightweight deep neural network model for detection of COVID-19 using X-ray images. Int. J. Imaging Syst. Technol..

[175] Podder P., Das S.R., Mondal M.R.H., Bharati S., Maliha A., Hasan M.J., Piltan F. (2023). LDDNet: A Deep Learning Framework for the Diagnosis of Infectious Lung Diseases. Sensors.

[176] Hamad Q.S., Samma H., Suandi S.A. (2023). Feature selection of pre-trained shallow CNN using the QLESCA optimizer: COVID-19 detection as a case study. Appl. Intell..

[177] Asraf A., Islam Z. COVID19, Pneumonia and Normal Chest X-ray PA Dataset. 2021, Version 1. https://www.mdpi.com/1424-8220/23/1/480.

[178] Wahid F., Azhar S., Ali S., Zia M.S., Abdulaziz Almisned F., Gumaei A. (2022). Pneumonia Detection in Chest X-Ray Images Using Enhanced Restricted Boltzmann Machine. J. Healthc. Eng..

[179] COVID-19 Radiography Database. https://www.kaggle.com/datasets/tawsifurrahman/covid19-radiography-database.

[180] Visuña L., Yang D., Garcia-Blas J., Carretero J. (2022). Computer-aided diagnostic for classifying chest X-ray images using deep ensemble learning. BMC Med. Imaging.

[181] Lasker A., Ghosh M., Obaidullah S.M., Chakraborty C., Roy K. (2023). LWSNet—A novel deep-learning architecture to segregate Covid-19 and pneumonia from X-ray imagery. Multimed. Tools Appl..

[182] Danilov V.V., Litmanovich D., Proutski A., Kirpich A., Nefaridze D., Karpovsky A., Gankin Y. (2022). Automatic scoring of COVID-19 severity in X-ray imaging based on a novel deep learning workflow. Sci. Rep..

[183] Shazia A., Xuan T.Z., Chuah J.H., Usman J., Qian P., Lai K.W. (2021). A comparative study of multiple neural network for detection of COVID-19 on chest X-ray. EURASIP J. Adv. Signal Process..

[184] Ferreira J.R., Armando Cardona Cardenas D., Moreno R.A., de Fátima de Sá Rebelo M., Krieger J.E., Antonio Gutierrez M. Multi-View Ensemble Convolutional Neural Network to Improve Classification of Pneumonia in Low Contrast Chest X-Ray Images. Proceedings of the 2020 42nd Annual International Conference of the IEEE Engineering in Medicine & Biology Society (EMBC).

[185] Chharia A., Upadhyay R., Kumar V., Cheng C., Zhang J., Wang T., Xu M. (2022). Deep-Precognitive Diagnosis: Preventing Future Pandemics by Novel Disease Detection With Biologically-Inspired Conv-Fuzzy Network. IEEE Access.

[186] Nahiduzzaman, Goni O.F., Anower S., Islam R., Ahsan M., Haider J., Gurusamy S., Hassan R., Islam R. (2021). A Novel Method for Multivariant Pneumonia Classification Based on Hybrid CNN-PCA Based Feature Extraction Using Extreme Learning Machine With CXR Images. IEEE Access.

[187] Yi R., Tang L., Tian Y., Liu J., Wu Z. (2023). Identification and classification of pneumonia disease using a deep learning-based intelligent computational framework. Neural Comput. Appl..

[188] Aljawarneh S.A., Al-Quraan R. (2023). Pneumonia Detection Using Enhanced Convolutional Neural Network Model on Chest X-Ray Images. Big Data.

[189] Alshmrani G.M.M., Ni Q., Jiang R., Pervaiz H., Elshennawy N.M. (2023). A deep learning architecture for multi-class lung diseases classification using chest X-ray (CXR) images. Alex. Eng. J..

[190] Sanghvi H.A., Patel R.H., Agarwal A., Gupta S., Sawhney V., Pandya A.S. (2023). A deep learning approach for classification of COVID and pneumonia using DenseNet-201. Int. J. Imaging Syst. Technol..

[191] Ukwuoma C.C., Qin Z., Belal Bin Heyat M., Akhtar F., Bamisile O., Muaad A.Y., Addo D., Al-antari M.A. (2023). A hybrid explainable ensemble transformer encoder for pneumonia identification from chest X-ray images. J. Adv. Res..

[192] Ravi V., Acharya V., Alazab M. (2023). A multichannel EfficientNet deep learning-based stacking ensemble approach for lung disease detection using chest X-ray images. Clust. Comput..

[193] Erdogan Yildirim A., Canayaz M. (2023). A novel deep learning-based approach for prediction of neonatal respiratory disorders from chest X-ray images. Biocybern. Biomed. Eng..

[194] Bal U., Bal A., Moral Ö.T., Düzgün F., Gürbüz N. (2024). A deep learning feature extraction-based hybrid approach for detecting pediatric pneumonia in chest X-ray images. Phys. Eng. Sci. Med..

[195] Chen S., Ren S., Wang G., Huang M., Xue C. (2024). Interpretable CNN-Multilevel Attention Transformer for Rapid Recognition of Pneumonia from Chest X-Ray Images. IEEE J. Biomed. Health Inform..

[196] Simonyan K., Zisserman A. (2015). Very Deep Convolutional Networks for Large-Scale Image Recognition. arXiv.

[197] Deng J., Dong W., Socher R., Li L.-J., Li K., Fei-Fei L. ImageNet: A large-scale hierarchical image database. Proceedings of the 2009 IEEE Conference on Computer Vision and Pattern Recognition.

[198] Selvaraju R.R., Cogswell M., Das A., Vedantam R., Parikh D., Batra D. (2020). Grad-CAM: Visual Explanations from Deep Networks via Gradient-Based Localization. Int. J. Comput. Vis..

[199] Garcia Santa Cruz B., Bossa M.N., Sölter J., Husch A.D. (2021). Public COVID-19 X-ray datasets and their impact on model bias–A systematic review of a significant problem. Med. Image Anal..

[200] Zunaed M., Haque A., Hasan T. (2024). Learning to Generalize Towards Unseen Domains via a Content-Aware Style Invariant Model for Disease Detection From Chest X-rays. IEEE J. Biomed. Health Inform..

[201] Horry M.J., Chakraborty S., Pradhan B., Paul M., Zhu J., Loh H.W., Barua P.D., Acharya U.R. (2023). Development of Debiasing Technique for Lung Nodule Chest X-ray Datasets to Generalize Deep Learning Models. Sensors.

[202] Arias-Garzón D., Tabares-Soto R., Bernal-Salcedo J., Ruz G.A. (2023). Biases associated with database structure for COVID-19 detection in X-ray images. Sci. Rep..

[203] Afshar P., Heidarian S., Naderkhani F., Oikonomou A., Plataniotis K.N., Mohammadi A. (2020). COVID-CAPS: A capsule network-based framework for identification of COVID-19 cases from X-ray images. Pattern Recognit. Lett..

[204] Pereira R.M., Bertolini D., Teixeira L.O., Silla C.N., Costa Y.M.G. (2020). COVID-19 identification in chest X-ray images on flat and hierarchical classification scenarios. Comput. Methods Programs Biomed..

[205] Jia L.-L., Zhao J.-X., Pan N.-N., Shi L.-Y., Zhao L.-P., Tian J.-H., Huang G. (2022). Artificial intelligence model on chest imaging to diagnose COVID-19 and other pneumonias: A systematic review and meta-analysis. Eur. J. Radiol. Open.

[206] Aslani S., Jacob J. (2023). Utilisation of deep learning for COVID-19 diagnosis. Clin. Radiol..

[207] Wang Z., Xiao Y., Li Y., Zhang J., Lu F., Hou M., Liu X. (2021). Automatically discriminating and localizing COVID-19 from community-acquired pneumonia on chest X-rays. Pattern Recognit..

[208] Gupta A., Anjum, Gupta S., Katarya R. (2021). InstaCovNet-19: A deep learning classification model for the detection of COVID-19 patients using Chest X-ray. Appl. Soft Comput..

[209] Brunese L., Mercaldo F., Reginelli A., Santone A. (2020). Explainable Deep Learning for Pulmonary Disease and Coronavirus COVID-19 Detection from X-rays. Comput. Methods Programs Biomed..

[210] Nazir S., Dickson D.M., Akram M.U. (2023). Survey of explainable artificial intelligence techniques for biomedical imaging with deep neural networks. Comput. Biol. Med..

[211] Jangam E., Annavarapu C.S.R., Barreto A.A.D. (2023). A multi-class classification framework for disease screening and disease diagnosis of COVID-19 from chest X-ray images. Multimed. Tools Appl..

[212] Ong J.H., Goh K.M., Lim L.L. Comparative Analysis of Explainable Artificial Intelligence for COVID-19 Diagnosis on CXR Image. Proceedings of the 2021 IEEE International Conference on Signal and Image Processing Applications (ICSIPA).

[213] Karim R., Döhmen T., Cochez M., Beyan O., Rebholz-Schuhmann D., Decker S. DeepCOVIDExplainer: Explainable COVID-19 Diagnosis from Chest X-ray Images. Proceedings of the 2020 IEEE International Conference on Bioinformatics and Biomedicine (BIBM).

[214] Guidotti R., Monreale A., Ruggieri S., Turini F., Giannotti F., Pedreschi D. (2018). A Survey of Methods for Explaining Black Box Models. ACM Comput. Surv..

[215] Gillmann C., Smit N.N., Gröller E., Preim B., Vilanova A., Wischgoll T. (2021). Ten Open Challenges in Medical Visualization. IEEE Comput. Graph. Appl..

[216] Nour M., Cömert Z., Polat K. (2020). A Novel Medical Diagnosis model for COVID-19 infection detection based on Deep Features and Bayesian Optimization. Appl. Soft Comput..

[217] Li Z., Kamnitsas K., Glocker B., Shen D., Liu T., Peters T.M., Staib L.H., Essert C., Zhou S., Yap P.-T., Khan A. (2019). Overfitting of Neural Nets Under Class Imbalance: Analysis and Improvements for Segmentation. Proceedings of the Medical Image Computing and Computer Assisted Intervention–MICCAI 2019.

[218] Fernández A., García S., Herrera F., Corchado E., Kurzyński M., Woźniak M. (2011). Addressing the Classification with Imbalanced Data: Open Problems and New Challenges on Class Distribution. Proceedings of the Hybrid Artificial Intelligent Systems.

[219] Hertel R., Benlamri R. (2023). Deep Learning Techniques for COVID-19 Diagnosis and Prognosis Based on Radiological Imaging. ACM Comput. Surv..

[220] Rajaraman S., Siegelman J., Alderson P.O., Folio L.S., Folio L.R., Antani S.K. (2020). Iteratively Pruned Deep Learning Ensembles for COVID-19 Detection in Chest X-rays. IEEE Access.

[221] Alom M.Z., Rahman M.M.S., Nasrin M.S., Taha T.M., Asari V.K. (2020). COVID_MTNet: COVID-19 Detection with Multi-Task Deep Learning Approaches. arXiv.

[222] Khattab R., Abdelmaksoud I.R., Abdelrazek S. (2024). Automated detection of COVID-19 and pneumonia diseases using data mining and transfer learning algorithms with focal loss from chest X-ray images. Appl. Soft Comput..

[223] Siddiqi R., Ahraf S.N., Kandhro I.A. (2023). Susceptibility of paediatric pneumonia detection model under projected gradient descent adversarial attacks. Int. J. Electron. Secur. Digit. Forensics.

[224] Kaviani S., Han K.J., Sohn I. (2022). Adversarial attacks and defenses on AI in medical imaging informatics: A survey. Expert Syst. Appl..

[225] Asgari Taghanaki S., Das A., Hamarneh G., Stoyanov D., Taylor Z., Kia S.M., Oguz I., Reyes M., Martel A., Maier-Hein L., Marquand A.F., Duchesnay E., Löfstedt T. (2018). Vulnerability Analysis of Chest X-ray Image Classification Against Adversarial Attacks. Understanding and Interpreting Machine Learning in Medical Image Computing Applications.

[226] Xu M., Zhang T., Zhang D. (2022). MedRDF: A Robust and Retrain-Less Diagnostic Framework for Medical Pretrained Models Against Adversarial Attack. IEEE Trans. Med. Imaging.

[227] Kong F., Liu F., Xu K., Shi X. (2023). Why does batch normalization induce the model vulnerability on adversarial images?. World Wide Web.

[228] Das D., Biswas S.K., Bandyopadhyay S. (2022). Perspective of AI system for COVID-19 detection using chest images: A review. Multimed. Tools Appl..

[229] Paschali M., Conjeti S., Navarro F., Navab N., Frangi A.F., Schnabel J.A., Davatzikos C., Alberola-López C., Fichtinger G. (2018). Generalizability vs. Robustness: Investigating Medical Imaging Networks Using Adversarial Examples. Proceedings of the Medical Image Computing and Computer Assisted Intervention–MICCAI 2018.

[230] Ma X., Niu Y., Gu L., Wang Y., Zhao Y., Bailey J., Lu F. (2021). Understanding adversarial attacks on deep learning based medical image analysis systems. Pattern Recognit..

[231] Hirano H., Minagi A., Takemoto K. (2021). Universal adversarial attacks on deep neural networks for medical image classification. BMC Med. Imaging.

[232] Moosavi-Dezfooli S.-M., Fawzi A., Fawzi O., Frossard P. (2017). Universal Adversarial Perturbations. Proceedings of the 2017 IEEE Conference on Computer Vision and Pattern Recognition (CVPR).

[233] Dai Y., Qian Y., Lu F., Wang B., Gu Z., Wang W., Wan J., Zhang Y. (2023). Improving adversarial robustness of medical imaging systems via adding global attention noise. Comput. Biol. Med..

[234] Sheikh B.U.H., Zafar A. (2024). Removing Adversarial Noise in X-ray Images via Total Variation Minimization and Patch-Based Regularization for Robust Deep Learning-based Diagnosis. J. Imaging Inform. Med..

